# Entropy Bounds and Capacity-Limited Information Flow in Black-Hole Evaporation

**DOI:** 10.3390/e28060671

**Published:** 2026-06-11

**Authors:** Arkady Bolotin

**Affiliations:** Ben-Gurion University of the Negev, P.O. Box 653, Beersheba 8410501, Israel; arkadyv@bgu.ac.il

**Keywords:** entropy bounds, information flow, black-hole evaporation, quantum entanglement, Page curve, unitary evolution, information-transfer capacity

## Abstract

Black-hole evaporation exhibits a range of characteristic entropic phenomena, including Hawking thermality, monotonically increasing radiation entropy in semiclassical treatments, and the Page-curve behavior required by unitarity. These features are accompanied by long-standing puzzles concerning information loss, entanglement growth, and the transfer of correlations between a black hole and its radiation. In this work we present an information-theoretic analysis of these phenomena based on a discrete causal model in which entropy evolution is governed by a competition between the growth of accessible degrees of freedom and a finite capacity for transmitting correlations across a boundary. Radiation is generated through stochastic sampling of interior degrees of freedom, while entanglement between interior and radiation subsystems is constrained by a boundary defined purely at the level of causal connectivity. Within this setting, radiation entropy increases at early times, reaches a maximum when boundary capacity becomes saturated, and decreases thereafter as additional emissions fail to carry independent correlations, yielding Page-curve behavior consistent with unitary evaporation. This capacity-limited mechanism does not rely on semiclassical spacetime geometry or quantum extremal surface constructions and instead follows directly from entropy bounds and information-flow constraints. By isolating the role of finite correlation capacity, the analysis provides a unified entropy-based perspective on black-hole evaporation, complementing semiclassical approaches while remaining applicable in discrete or non-geometric settings.

## 1. Introduction

Black-hole evaporation is characterized by several interrelated entropic phenomena. Semiclassically, Hawking radiation is thermal and leads to a steady increase in the radiation entropy, apparently implying information loss. However, unitarity requires that the radiation entropy eventually decrease, producing the Page curve [1,2]. Reconciling these features has given rise to a range of conceptual puzzles involving entanglement growth, information transfer between a black hole and its radiation, and the role of interior degrees of freedom.

A variety of approaches have been developed to address these tensions. Semiclassical studies of Hawking radiation [3] and quantum fields in curved spacetime [4] introduced the thermality puzzle. Holographic and quantum information-theoretic developments [5,6]—including black-hole complementarity [7], the firewall paradox [8], holographic entanglement entropy [9,10,11], and the quantum extremal surface (QES) program [12,13]—demonstrated how semiclassical predictions fail once quantum correlations are tracked consistently. Causal-structure approaches, such as causal sets [14,15] and quantum causal models [16,17], underscored the importance of discrete connectivity and nonlocal informational constraints in regimes where geometry is not fundamental.

In this work we adopt a complementary, entropy-centered perspective. Rather than focusing on geometric optimization, we examine how entropy production, saturation, and purification arise from information-flow constraints in a discrete causal model. Radiation is generated through stochastic sampling of interior degrees of freedom, while correlations between interior and radiation subsystems are constrained by a boundary defined purely in terms of causal connectivity. This setup allows entropy bounds and information-transfer capacity to be formulated without reference to semiclassical spacetime geometry.

Within this framework, the Page curve arises naturally as one manifestation of a capacity-limited entropic process. Radiation entropy initially increases as new degrees of freedom become accessible, reaches a maximum when the boundary’s correlation capacity is saturated, and decreases thereafter as further emissions fail to transmit independent correlations. The Page time is identified as the crossover between content-driven entropy growth and capacity-limited entropy saturation.

The aim of this work is to clarify the entropy-based mechanisms underlying black-hole evaporation and its associated paradoxes. By focusing on capacity constraints and information flow, we provide a unifying entropy-theoretic description that complements semiclassical treatments while remaining applicable in discrete or non-geometric settings.

## 2. Discrete Causal–Informational Setup

In this section, we present a minimal information-theoretic setup—partly introduced in [18,19]—sufficient to analyze entropy growth, saturation, and purification in black-hole evaporation.

**Axiom** **1** (Discreteness and Local Finiteness of Hodons)**.** *The fundamental description of physical processes involves a countable set P of elementary events called hodons. For every operationally defined subsystem R⊆P obtained by coarse-graining or restricting attention to a finite-resolution observation, the number of hodons in R is finite:*

|R|<∞.

*Thus, the hodon substrate is intrinsically discrete and locally finite, independently of any geometric interpretation.*

**Remark** **1** (Operational Meaning and Necessity of Finiteness)**.** *The terms “discrete” and “locally finite” in this axiom refer to the operational structure of the hodon set P: discreteness means that hodons are distinguishable informational events, and local finiteness means that any subsystem singled out by a finite-resolution observational or coarse-graining procedure contains only finitely many such events. Although this may appear automatic from the notion of finite resolution, the axiom serves an essential foundational role: without explicitly requiring that every operationally defined subset be finite, one could encounter pathological cases in which a coarse-grained or finite-resolution operation selects infinitely many hodons (for example, when infinitely many events are indistinguishable at the chosen resolution). Thus, Axiom 1 imposes a necessary constraint on the underlying substrate, ensuring that the hodon framework remains mathematically coherent and operationally meaningful at all scales.*

**Axiom** **2** (Informational Structure of Hodons)**.** *Each hodon p∈P possesses a finite dimensional Hilbert space H(p) representing its intrinsic informational degrees of freedom. Physical observables associated with a hodon act on this local Hilbert space, and correlations between hodons are mediated by link degrees of freedom.*

**Axiom** **3** (Poisson Statistics of Hodon Distribution)**.** *For any finite-resolution selection procedure S that operationally identifies a subset of hodons, the number of selected hodons*

N(S):=|P(S)|

*is a Poisson-distributed random variable,*

$$P\;\left(N(S)=n\right)=\frac{\lambda {\left(S\right)}^{n}}{n!}\;e^{-\lambda (S)},\;\;n\in N.$$

*Here, λ(S)=αμ(S) for a constant α and an abstract additive measure μ on such selection procedures. In any continuum approximation, μ maps to spacetime volume under a continuum approximation functor F.*

**Remark** **2** (Selection Procedure)**.** *In Axiom 3, a selection procedure S is a finite-resolution operation that determines which hodons are detected or coarse-grained. It produces a finite subset P(S)⊆P, whose size N(S) is Poisson distributed.*

**Axiom** **4** (QHIS)**.** *A quantum hodon incidence structure (QHIS, for short) is a tuple*

Q=(P,L,I,W,H)

*consisting of:*

*(**i**) **Finite event structure.*** *A finite set P of hodons (distinguishable events), and a finite set L of directed links. Each link ℓ∈L has a specified source s(ℓ)∈P and target t(ℓ)∈P.*
*(**ii**) **Incidence relation.*** *An incidence relation I⊆(P×L)∪(L×P) satisfying: s(ℓ)Iℓ, ℓIt(ℓ), and no other incidences. This induces a directed adjacency structure p≺q if there exists ℓ∈L with s(ℓ)=p and t(ℓ)=q.*
*(**iii**) **Probabilistic causal weights.*** *A weight assignment*
  (1) W:L→[0,1]
  *such that for every p∈P,*
  $$\sum_{\begin{array}{c} \ell \in L\\ s(\ell )=p \end{array}}W\left(\ell \right)\;\leq \;1.\;$$
  *The weight W(ℓ) is interpreted as an operational propensity for influence to propagate from s(ℓ) to t(ℓ).*
*(**iv**) **Path composition and reachability.** For any directed path $\gamma =\ell_{1}\ell_{2}\ldots \ell_{k}$ with $t\left(\ell_{i}\right)=s\left(\ell_{i+1}\right)$, define its weight*
  $$W\left(\gamma \right):=\prod_{i=1}^{k}W\left(\ell_{i}\right),\;$$
  *and define the reachability from p to q as*
  $$\Pi \left(p⇝q\right):=\underset{\gamma \in \Gamma (p,q)}{\sup}W\left(\gamma \right),\;\sup⌀=0,\;$$
  *where Γ(p,q) is the set of directed paths from p to q.*

**Remark** **3** (Subnormalized Outgoing Propensities)**.** *The condition (1) does not require the outgoing weights at a hodon p to form a normalized probability distribution. The map W assigns propensities for influence to propagate along explicitly represented links, while the deficit $1-\sum_{s(l)=p}W\left(l\right)$ corresponds to the possibility that no further influence is transmitted (e.g. absorption, termination, or propagation along unresolved coarse-grained channels). Thus, a QHIS need not be stochastically complete at each node; only the existence and relative strengths of the modeled influence channels are encoded in W.*

**Remark** **4** (Zero-Weight Links and Effective Pruning)**.** *A link l∈L with W(l)=0 is combinatorially present in the incidence structure but carries no admissible causal influence: no path of positive weight can make essential use of l. Operationally, such a link is indistinguishable, for information-flow purposes, from the absence of a link between its endpoints. One may therefore define an effective link set*

$$L_{\mathrm{eff}}:=\left\{\;l\in L|W\left(l\right)>0\;\right\},$$

*obtained by pruning all zero-weight links. This pruning leaves the reachability relation Π(p⇝q) and all subsequent constructions based on positive-propensity paths unchanged.*

**Remark** **5** (Proposition 1 and Zero-Weight Pruning)**.** *Proposition 1 remains valid if one replaces L by the pruned set $L_{\mathrm{eff}}$ that discards all links with W(l)=0. Since zero-weight links do not contribute to positive-propensity paths, the causal skeleton*

$$(P,L_{\mathrm{eff}},I{|}_{L_{\mathrm{eff}}},W{|}_{L_{\mathrm{eff}}})\;$$

*has the same operational reachability structure as (P,L,I,W). Consequently, the canonical lifting to a QHIS and the restriction back to the causal skeleton are insensitive to whether zero-weight links are retained or removed; the two descriptions differ only by the presence or absence of causally inactive edges.*

*(**v**) **Local finiteness and acyclicity.*** *There are no directed cycles supported by positive-propensity paths: Π(p⇝p)=0 for all p∈P. Each hodon has only finitely many predecessors and successors under* Π.
*(**vi**) **Hilbert assignments.** A Hilbert space assignment*
(2) H:P∪L→Hilb,
  *where Hilb denotes the class of finite dimensional Hilbert spaces required by Axiom 2. This assignment associates to each hodon and each link the local quantum degrees of freedom carried by events and influence channels within the structure.*

**Remark** **6** (Relation to Axiom 2 and the Role of H)**.** *Axiom 2 should be distinguished from clause (**vi**). Axiom 2 provides the physical justification for employing finite dimensional Hilbert spaces by asserting that each hodon carries a finite informational state space. Clause (**vi**), by contrast, formally incorporates the map (2) into the definition of a QHIS. Thus, Axiom 2 specifies what the relevant Hilbert spaces are required to be, while clause (**vi**) specifies how they are built into the structure of the QHIS itself.*

*(**vii**) **Classicalization regime.** There exists a coarse-graining/decoherence map under which W(ℓ)∈{0,1} for all ℓ∈L, and the nodal and link Hilbert spaces in H collapse to minimal carriers, so that the induced relation* ≺ *becomes a partial order.*
*(**viii**) **Incidence boundary for subsystems.** For any operationally defined subset R⊆P of hodons, an incidence boundary ∂R is any minimal set of links whose removal eliminates all positive-propensity paths (i.e., paths with nonzero weight under W) between R and its complement P∖R in both directions. This notion of boundary is purely combinatorial and defined without reference to geometric structure.*

**Definition** **1** (Causal Consistency (Additional Structural Assumption))**.** *A QHIS is said to be causally consistent if (i) correlations may propagate only along those links ℓ∈L whose source and target are specified by the incidence relation I—that is, no influence is permitted except through links ℓ∈L with specified source s(ℓ) and target t(ℓ)—and (ii) the evolution of the global quantum state ρ, a density operator on*

(3) $$H_{\mathrm{global}}=\underset{p\in P}{⨂}H_{P}\left(p\right)\;⊗\;\underset{\ell \in L}{⨂}H_{L}\left(\ell \right),\;$$

*is unitary in a manner compatible with the directed structure encoded by L. This condition ensures that the probabilistic and quantum enrichments introduced in Axiom 4 are used in a way that respects the operational notion of causality determined by the incidence structure.*

**Remark** **7** (Relation Between Causal Weights and Hilbert Assignments)**.** *In a QHIS tuple Q=(P,L,I,W,H), the causal-weight map (1) and the Hilbert assignment (2) serve distinct foundational roles. The map W is the primitive carrier of causal structure: it determines which influence channels exist and with what probabilistic strength, and it is from W that both the classical partial order and the emergent continuum geometry ultimately arise. By contrast, H equips each hodon and link with finite-dimensional quantum degrees of freedom, specifying the informational content that can, in principle, be transmitted.*

*Although Axiom 4 treats W and H as independent inputs, they are related structurally rather than algebraically. First, W determines where the Hilbert spaces associated with nodes and links may interact: if W(ℓ)=0, no causal influence is permitted across ℓ, regardless of the dimension of H(ℓ). Second, the magnitudes and distribution of the weights W(ℓ) bound the effective information-carrying capacity of the network, constraining how quantum correlations encoded in H may propagate. Third, in the classicalization regime of clause (**vii**), deterministic causal weights coincide with the collapse of the local Hilbert spaces to classical informational carriers, so the reductions in W and H occur in tandem.*

*Thus, while there is no direct functional dependence between W and H in the axiomatic specification of a QHIS, the two structures are operationally inseparable in determining how information flows through the network, how geometry emerges, and how geometric breakdown affects quantum correlations.*

**Axiom** **5** (Holographic Counting Bound)**.** *For any operationally defined subset R⊆P of hodons, let ∂R denote its incidence boundary as defined in clause (**viii**) of Axiom 4. The maximal informational capacity accessible to observers restricted to R—denoted I(R) (e.g., a supremum of von Neumann entropies over operational coarse-grainings compatible with the incidence constraints)—is bounded by*

I(R)≤βν(∂R),

*where ν(∂R) is an abstract measure defined on the incidence boundary, satisfying monotonicity and finite additivity. The constant β is the holographic density coefficient, representing the amount of information per unit of this abstract boundary measure.*

*In the continuum approximation, the functor F maps ν(∂R) to the geometric area Area(∂R), and β assumes its familiar value so that I(R) reproduces the standard holographic area law (e.g., $I\left(R\right)\leq A\left(\partial R\right)/\left(4\ell_{P}^{2}\right)$ up to units). Thus, the axiom is fully operational and remains geometry-agnostic at the fundamental level.*

### Philosophical Commentary: Operationalism and the Hodon Axioms

The axioms of the hodon framework align naturally with an operationalist philosophy of physics, in which the fundamental structures of a theory are grounded in what observers can, in principle, distinguish, register, or manipulate. Rather than positing a pre-existing spacetime manifold, the framework begins with the minimal ingredients needed to describe observable events, informational content, and causal influence. Geometry, in this view, is an emergent descriptor that arises only after appropriate coarse-graining and regularity conditions are satisfied.

The *Discreteness and Local Finiteness Axiom* (1) expresses the idea that the fundamental substrate consists of distinguishable informational events (hodons), and that any operationally defined subsystem contains only finitely many such events. This is not a geometric assumption but an operational one: no measurement procedure can isolate infinitely many distinguishable events within finite resolution. Thus, “discreteness” refers to the distinguishability of hodons, and “local finiteness” refers to the finite informational content accessible in any operationally defined subset, without invoking metric or topological notions.

The *Informational Nature of Hodons Axiom* (2) develops this operational viewpoint. Rather than treating spacetime points or metric intervals as primitive, the framework posits that the basic carriers of physical information are the hodons themselves, each equipped with a finite-dimensional Hilbert space. All observables—geometric, dynamical, or thermodynamic—are ultimately emergent functionals of the informational degrees of freedom and correlations encoded in these Hilbert spaces and the links between them. The axiom embodies a shift from a geometry-first ontology to an information-first ontology.

The *Poisson Statistics Axiom* (3) introduces a statistical regularity for the outcomes of finite-resolution selection procedures. Instead of assigning a fixed cardinality to a pre-specified domain, the axiom treats the number of hodons identified by any such operational procedure as a Poisson-distributed random variable with intensity λ(S)=αμ(S). Here, μ is an abstract additive measure assigned to selection procedures rather than to geometric regions, providing a primitive notion of “sampling extent” entirely independent of metric or topological structure. In simple terms, the axiom characterizes how many hodons one can expect to “pull out” using a given finite-resolution selection, and how strongly that count fluctuates due to the intrinsic randomness of the elementary events.

The *QHIS Axiom* (4) introduces the core structure of the hodon framework: a QHIS tuple (P,L,I,W,H) combining discrete events, directed links, probabilistic causal propensities, and finite-dimensional Hilbert-space assignments. The incidence relation *I* and the weights *W* jointly encode causal influence through the reachability relation of clause (**iv**). Acyclicity and local finiteness of influence (clause (**v**)) ensure that causal paradoxes do not arise at the discrete level. Clause (**vi**) specifies how Hilbert spaces are assigned to hodons and links within the QHIS tuple, thereby grounding the quantum degrees of freedom of the system. Classicalization (clause (**vii**)) describes the regime in which probabilistic and quantum enrichments collapse to yield a partial order suitable for geometric approximation. Finally, clause (**viii**) introduces the incidence boundary of an operationally defined subset, a purely combinatorial construct that later supports the holographic counting bound without presupposing geometric notions such as metric area.

The *Holographic Counting Axiom* (5) provides an informational bound on what can be operationally distinguished within any finite-resolution subset of hodons. As compared to Axiom 3, Axiom 5 bounds how much information can be accessed from the Poisson-sampled hodons P(S) produced by a finite-resolution selection procedure S. Simply put, the Poisson axiom describes *how many* hodons are likely to be identified by a given selection, while the holographic axiom describes *how much information* those hodons can support. For example, a large sampling extent μ(S) together with a small boundary measure ν(∂R), where R=P(S), indicates that the incidence boundary is too limited to support N(S) distinguishable informational degrees of freedom.

**Remark** **8** (Indispensability of Axiom 3)**.** *The Poisson axiom plays an important supporting role in the physical interpretation of the framework. First, it ensures a statistically homogeneous discreteness: hodons appear with well-controlled fluctuations under refinement, preventing artificial clustering or voids and enabling stable coarse-grained behavior. Second, it complements the Holographic Counting Axiom 5 by providing a natural “content vs. capacity” structure. Finally, Poisson fluctuations supply the statistical substrate needed to analyze transitions between geometric and non-geometric regimes. Thus, Axiom 3 is operationally indispensable: it furnishes the probabilistic structure required to interpret the hodon network as a physically coherent informational substrate.*

In sum, the hodon axioms articulate an operational and informational foundation for physical theory in which the basic ingredients are distinguishable events, their allowed causal influences, and the quantum informational content they carry. Nothing beyond these primitives is assumed. Higher-level structures—such as effective notions of continuity, extended degrees of freedom, or classical behavior—arise only when the probabilistic and informational patterns encoded in the hodon network exhibit sufficient regularity under coarse-graining. In this sense, the framework provides a minimal and internally consistent substrate from which the familiar structures of physics can emerge without being built in at the outset.

## 3. From Graphs to Incidence

This section clarifies the structural hierarchy that underlies the QHIS formalism. A QHIS contains quantum, probabilistic, and combinatorial data; by systematically forgetting these data in stages, we obtain a sequence of simpler objects. This hierarchy is essential for later discussions of classicalization, geometric realizability, and the conditions under which singular behavior arises.

**Definition** **2** (Underlying Probabilistically Causal Graph)**.** *Given a QHIS Q as in Axiom 4, its underlying probabilistically causal graph (or causal skeleton) is the* 4*-tuple*

(4) $$G\;:=\;\underline{Q}\;=\;\left(P,L,I,W\right),\;$$

*obtained by forgetting the Hilbert assignment H (2). Clauses (**i**)–(**v**) and (**viii**) of Axiom 4 apply verbatim to G.*

**Proposition** **1.** *Let Q be a QHIS in the sense of Axiom 4, and let G be its causal skeleton (4). Then, G canonically lifts to Q up to the choice of a Hilbert assignment (2). Conversely, every QHIS restricts to its causal skeleton by forgetting H. Thus,*

G⟷Q

*is one-to-one up to the choice of H.*

In this sense, the causal skeleton captures the purely relational backbone of a QHIS, while the Hilbert assignments supply its quantum degrees of freedom.

**Definition** **3** (Underlying Incidence Structure)**.** *Let G be a QHIS causal skeleton (4). Its underlying incidence structure is the* 3*-tuple*

(5) $$C\;:=\;\underline{G}\;=\;\left(P,L,I\right),\;$$

*obtained by forgetting the probabilistic weights W (1). Clauses (**i**) and (**ii**) of Axiom 4 apply to C.*

**Proposition** **2.** *Let G be a QHIS causal skeleton (4) with underlying incidence structure C (5). Then, G restricts to C by forgetting W.*

*Conversely, if C admits at least one assignment of orientations and weights (1), then each such choice yields a probabilistically causal graph (4) whose underlying incidence structure is C. Whenever such a lift exists,*

C⟷G

*is one-to-one up to admissible weight assignments W(ℓ).*

### Regularity and Manifold-Likeness Conditions

To determine when an incidence structure may support a continuum approximation, we introduce purely combinatorial criteria that capture when the pattern of incidences is sufficiently controlled. These conditions refer only to the data (P,L,I) arising in the classicalization limit and do not presuppose any geometric interpretation.

**Definition** **4** (Regularity Conditions)**.** *Let C be an incidence structure (5). We say that C satisfies the regularity conditions if:*

*(R1)*  ***Coherent adjacency:** Refinements of operational coarse-grainings of P modify the incidence pattern only locally, without introducing large or discontinuous changes.*
*(R2)*  ***Stability under refinement:** Successive refinements of P eventually stabilize in the sense that further refinement changes incidences only within bounded regions.*
*(R3)*  ***Causal compatibility:** The classicalized partial order* ≺ *is respected under refinement; if p≺q, then incidences between sufficiently refined coarse-grained subsets containing p and q are compatible with this order.*

**Definition** **5** (Manifold-Likeness Criteria)**.** *An incidence structure C is manifold-like if, in addition to the regularity conditions, it satisfies:*

*(M1)*  ***Coherent local patterns:** Around each p∈P, the induced subgraph on any sufficiently small coarse-graining cell containing p is isomorphic to one of a fixed finite family of model local patterns.*
*(M2)*  *=**Consistent scaling behavior:** Under successive refinements, these model patterns evolve in a controlled manner, enabling the continuum functor F to assign consistent continuum neighborhoods.*

These conditions characterize when an incidence structure behaves coherently under refinement, allowing it to serve as the discrete substrate for a continuum approximation. They play a central role in determining which classicalized QHIS configurations admit geometric realizations and which lead instead to non-geometric or singular phases.

In summary, the QHIS formalism organizes relational information in a layered form: the incidence structure (P,L,I) encodes the fundamental event–link relations; probabilistic weights refine this with causal intensities; and Hilbert assignments supply quantum informational content. Later sections build on this hierarchy to formulate the classicalization and geometrization theorems and to analyze the structural origin of gravitational singularities.

**Remark** **9** (Terminology)**.** *Throughout the manuscript, the abbreviation QHIS is used in two closely related senses: (i) a specific mathematical object Q=(P,L,I,W,H), and (ii) the broader QHIS framework, the theoretical setting in which such structures are defined and studied. The intended meaning is always clear from context.*

## 4. When a Classical Incidence Structure Is Recoverable from a QHIS

A QHIS reduces to a classical incidence structure precisely in the classicalization regime, where the probabilistic and quantum enrichments degenerate to a deterministic causal pattern.

**Theorem** **1** (QHIS Classical Limit)**.** *Let Q be a QHIS in the sense of Axiom 4. If clause (**vii**) (the classicalization regime) applies—so that W(ℓ)∈{0,1} for all ℓ∈L and the induced relation* ≺ *becomes a partial order—then the probabilistically causal graph (4) admits a well-defined incidence structure (5). In this regime, the probabilistic and quantum data collapse to a deterministic causal incidence structure.*

**Proof.**  Under clause (**vii**) of Axiom 4, the classicalization regime provides a coarse-graining or decoherence map such that all probabilistic weights collapse to W(ℓ)∈{0,1} and the induced relation ≺ becomes a partial order on *P*. Applying the forgetful functor $\underline{\left(\cdot \right)}$—that is, discarding the probabilistic and quantum structure of the QHIS—yields the underlying probabilistically causal graph (4).

Since all weights are now deterministic and ≺ is a partial order, forgetting the weights produces a well-defined incidence structure (5). In this structure, the probabilistic and quantum data have collapsed, and the QHIS reduces to a deterministic causal incidence pattern. This establishes the classical limit. □

### Interpretation

Theorem 1 identifies the regime in which a QHIS behaves effectively as a deterministic causal structure. In physical terms, this corresponds to a regime in which

- Fluctuations in causal influence become negligible;
- Correlations behave classically rather than quantum-mechanically;
- The quantum degrees of freedom decohere into minimal informational carriers.

This classicalization limit furnishes a *deterministic causal precursor* of spacetime structure, but it is not yet geometric. Classicalization ensures only that the incidence relations are well defined and acyclic; additional combinatorial criteria are necessary before any continuum interpretation becomes meaningful. The subsequent section introduces precisely those criteria.

## 5. Classical Limit → Classical Geometric Regime

Starting from the deterministic causal incidence structure obtained in the classicalization regime (Theorem 1), the next question is when such a structure admits a meaningful continuum interpretation. Not every incidence pattern can serve as the discrete precursor of a spacetime; further combinatorial coherence is required. Section 3 introduced the regularity and manifold-likeness conditions (R1)–(R3) and (M1)–(M2), which ensure that incidence relations vary coherently under refinement and that their local patterns belong to a stable finite family. When these conditions hold, the continuum approximation functor F can consistently assign smooth continuum data to the discrete structure. Theorem 2 formalizes this geometric regime, showing that a smooth Lorentzian manifold arises when the underlying incidence structure satisfies these combinatorial coherence properties.

**Theorem** **2** (QHIS Classical Geometric Regime)**.** *Let Q be a QHIS, and assume the conditions of the classical limit in Theorem 1, so that the induced relation* ≺ *is a partial order and C is the underlying incidence structure (5). Let ${\mathrm{IncStruct}}_{\mathrm{geom}}$ denote the class of incidence structures that satisfy the regularity conditions (R1)–(R3) and the manifold-likeness criteria (M1)–(M2), and let Lor stand for the class of smooth Lorentzian spacetimes (M,g), optionally viewed as a category whose morphisms are causal or isometric embeddings. Then, the continuum approximation functor*

(6) $$F:{\mathrm{IncStruct}}_{\mathrm{geom}}\to \mathrm{Lor}\;$$

*assigns to C a smooth Lorentzian spacetime (M,g) and an embedding ι:P↪M such that:*

*1.*  *The causal order induced by (M,g) restricts to the partial order* ≺ *on P;*
*2.*  *The image ι(P) reproduces, under coarse-graining, the adjacency and incidence relations encoded by C;*
*3.*  *ι(P) is sufficiently rich to generate (M,g) as the continuum approximation of Q.*

*In this regime, the QHIS admits a continuum description in terms of a smooth Lorentzian manifold.*

**Proof.**  By Theorem 1, the classicalization regime of clause (**vii**) of Axiom 4 collapses the probabilistic and quantum data of Q to a deterministic incidence structure (5) equipped with a partial order ≺. This incidence structure provides the discrete relational backbone for any continuum interpretation.

Assume now that C satisfies the regularity conditions (R1)–(R3) and the manifold-likeness criteria (M1)–(M2) introduced in Section 3. These combinatorial conditions ensure that the incidence pattern varies coherently under refinement, that local patterns are drawn from a fixed family of stable models, and that the partial order ≺ is compatible with these local structures. Under these circumstances, the continuum approximation functor (6) can consistently assign continuum neighborhoods to coarse-grained subsets of *P* and extend this assignment to a smooth Lorentzian manifold (M,g).

By construction, the embedding ι:P↪M preserves the partial order ≺ as the restriction of the causal order on (M,g). Furthermore, the combinatorial patterns encoded by C are reproduced, under coarse-graining, by the adjacency structure of ι(P) in *M*. The manifold-likeness criteria guarantee that ι(P) is dense enough, in the coarse-grained sense, to generate (M,g) as the continuum approximation of Q.

Thus, whenever the incidence structure satisfies the regularity and manifold-likeness conditions, the QHIS lies in the classical geometric regime and admits a continuum interpretation as a smooth Lorentzian spacetime. □

**Remark** **10** (Analogy with Classical Incidence Geometry)**.** *Theorem 2 parallels the classical realizability problem of incidence geometry, where one asks whether a finite incidence configuration (P,L,I) can be represented within a given geometric category, such as points and lines in a plane. There, algebraic or combinatorial constraints may obstruct realization over $Q^{2}$ or $R^{2}$. Likewise, in the hodon framework, the combinatorial criteria (R1)–(R3) and (M1)–(M2) determine whether an incidence pattern is coherent enough for realization by the continuum functor F (6). The theorem thus plays a role analogous to a realizability criterion, distinguishing configurations that admit a smooth continuum interpretation from those that remain purely combinatorial.*

## 6. Singularity as Incidence Non-Realizability

Classicalization (Theorem 1) guarantees the existence of a deterministic causal incidence structure, but it imposes no constraints ensuring that this structure satisfies the regularity and manifold-likeness criteria (R1)–(R3) and (M1)–(M2). As a result, many incidence patterns produced by a QHIS will fall outside the geometric regime.

When an incidence structure fails these criteria, it becomes *non-realizable* by the continuum functor F (6): no smooth Lorentzian region can be assigned to it in a manner consistent with the causal order and adjacency relations. This marks a transition from a geometric to a non-geometric phase. In contexts such as black holes, such breakdowns naturally arise in interior subsets of hodons whose incidence relations become too irregular or crowded under refinement to admit a continuum interpretation.

Within the continuum description provided by F (6), the inability to extend the geometric structure across such a non-realizable incident subset is expressed as geodesic incompleteness. From the perspective of the hodon framework, however, this phenomenon is fundamentally combinatorial: singularity corresponds not to curvature divergence but to the failure of an incidence pattern to satisfy the structural conditions required for geometric realizability.

This section formalizes this correspondence. Theorem 3 shows that singular behavior in the continuum arises when the incidence structure of a region lies outside the geometric regime, and the subsequent corollaries develop the implications of this principle.

**Theorem** **3** (Singularity via Incidence Non-Realizability)**.** *Let Q be a QHIS and let R⊆P be any operationally defined subset with induced QHIS*

(7) $$Q_{R}:=\left(R,L_{R},I_{R},W_{R},H_{R}\right)\;$$

*and classical incidence structure*

(8) $$C\left(Q_{R}\right):=\left(R,L_{R},I_{R}\right).$$

*Suppose Q lies in the classical limit of Theorem 1 and that the continuum functor F (6) is defined. If $C(Q_{R})$ fails the geometric-regime criteria (R1)–(R3) and (M1)–(M2), then F cannot realize R as any smooth Lorentzian region; any continuum description attempting to extend smoothly across R is necessarily geodesically incomplete (i.e., singular).*

**Proof.**  By Theorem 1, in the classicalization regime, the probabilistic and quantum data collapse to a deterministic causal incidence structure on any operationally defined subset. By Theorem 2, the continuum functor (6) assigns a smooth Lorentzian region precisely to those incidence structures that satisfy (R1)–(R3) and (M1)–(M2). If $C(Q_{R})$ fails these criteria, then it lies outside the domain of realizability of F (6), so no smooth Lorentzian region can be assigned to *R*. Equivalently, any continuum description defined on the complement cannot be extended smoothly across *R*, i.e., the attempted extension is geodesically incomplete. Hence, *R* is singular in the continuum sense. □

We begin with the black-hole case as a concrete application of Theorem 3. It provides the most familiar physical example of the general principle that failure of the geometric-regime criteria forces any continuum realization to be singular.

**Corollary** **1** (Black-Hole Interior Singularity)**.** *Let Q be a QHIS and let $P_{\mathrm{int}}\subseteq P$ denote the operationally defined subset corresponding (under coarse-graining) to the interior region of a black hole. Assume the continuum approximation functor F (6) exists. Suppose the exterior sector lies in the geometric regime so that*

(M,g)=F(Q)

*realizes the exterior as a smooth Lorentzian spacetime. If the interior incidence structure $C(Q_{\mathrm{int}})$ fails the criteria (R1)–(R3) and (M1)–(M2), then the continuum description cannot be extended smoothly across the interior. The interior of (M,g) is therefore geodesically incomplete and singular.*

**Proof.**  Apply Theorem 3 to $R=P_{\mathrm{int}}$. The exterior sector is, by assumption, realized smoothly via F (6). Since $C(Q_{\mathrm{int}})$ fails the geometric-regime criteria, it is not realizable as a smooth Lorentzian region, and any attempted continuum extension across $P_{\mathrm{int}}$ is geodesically incomplete. Hence, the interior is singular. □

The following corollary states the general form of the singularity principle encapsulated in Theorem 3, showing that the black-hole interior is merely one instance of a universal phenomenon.

**Corollary** **2** (Breakdown of the Geometric Regime Implies Singularity)**.** *Let Q be a QHIS and let R⊆P be any operationally defined subset with induced QHIS (7) and classical incidence structure (8). If $C(Q_{R})$ fails the geometric-regime criteria (R1)–(R3) and (M1)–(M2), then any continuum description that would realize R is geodesically incomplete and thus singular. Conversely, if $C(Q_{R})$ satisfies these criteria and the hypotheses of Theorem 2 are obtained, then $F(Q_{R})$ exists and realizes R as a smooth, non-singular Lorentzian region.*

**Proof.**  The forward implication is an application of Theorem 3 to the subset *R*. For the converse, Theorem 2 ensures that if the geometric-regime criteria hold and its hypotheses are obtained, then F (6) realizes the incidence structure as a smooth Lorentzian region; smooth realizations are, by definition, non-singular. □

## 7. A Unified Framework: From Classicalization to Geometrization to Singularity Formation

The three theorems developed above form a coherent progression describing how structure emerges from a QHIS and how this structure may, or may not, support a continuum interpretation. Each theorem isolates a conceptually distinct stage, and together, they provide a layered account of how deterministic causality, continuum geometry, and singularity formation arise from the underlying quantum–informational substrate.

### 7.1. Theorem 1 (Classicalization)

The first stage is the collapse of probabilistic and quantum features. Under the classicalization regime of clause (**vii**) in Axiom 4, all weights become deterministic and the induced relation ≺ becomes a partial order. The QHIS therefore reduces to a deterministic causal incidence structure (P,L,I) in the sense of Definition 3. This represents the foundational relational backbone upon which all higher-level descriptions are built.

### 7.2. Theorem 2 (Geometrization)

Deterministic causality alone does not guarantee geometric interpretability. The incidence structure obtained from classicalization must also satisfy the regularity and manifold-likeness conditions (R1)–(R3) and (M1)–(M2) introduced in Section 3. These purely combinatorial conditions ensure that the incidence pattern behaves coherently under refinement and that local combinatorial patterns belong to a stable finite family. When they hold, the continuum approximation functor F (6) can consistently assign to the structure a smooth Lorentzian manifold, providing a valid continuum interpretation.

### 7.3. Theorem 3 (Singularity Formation)

The third stage concerns the breakdown of geometric realizability. If interior subsets of hodons fail to satisfy the regularity or manifold-likeness axioms, then the continuum functor cannot assign to these subsets any smooth Lorentzian region. Consequently, the continuum spacetime reconstructed from the exterior admits no smooth extension into the interior. Within the continuum description, this obstruction appears as geodesic incompleteness. Thus, singularity formation is not an additional physical postulate but the direct manifestation of incidence non-realizability.

### 7.4. Overview

**Figure 1:**
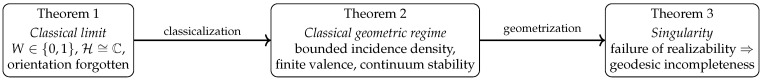
Logical flow from the classical limit of a QHIS (Theorem 1) to the classical geometric regime (Theorem 2), and finally, to singularity formation when realizability fails (Theorem 3).

Together, the three theorems articulate a unified and conceptually transparent framework for understanding the emergence and breakdown of continuum structure in QHIS. Quantum and probabilistic features collapse to yield deterministic causal order (Theorem 1); a smooth continuum geometry emerges only when this incidence pattern is sufficiently regular (Theorem 2); and when these regularity conditions are violated, the continuum description becomes incomplete and singular behavior appears (Theorem 3). This sequence captures a full structural lifecycle: from quantum discreteness, to classical causal structure, to continuum geometry when possible, and to the failure of geometry when the discrete structure departs from the geometric regime (see Figure 1).

## 8. Incidence Density as a Discrete Curvature Indicator

In the QHIS framework, curvature is not a primitive concept but an emergent descriptor introduced only after applying the continuum functor F (6). At the discrete level, any analog of curvature must therefore be encoded directly in the incidence structure (5) and in how this structure behaves under refinement. A useful combinatorial quantity capturing such behavior is *incidence density*, which measures the average number of links incident on elements of a refining sequence of finite subsets of *P*. While this quantity has no geometric meaning on its own, its divergence under refinement provides an operational signal that the corresponding incidence structure cannot belong to the manifold-like class characterized by the regularity and manifold-likeness criteria of Section 3.

### 8.1. Intuition

In incidence structures satisfying (R1)–(R3) and (M1)–(M2), combinatorial relations evolve in a stable fashion under refinement: local patterns remain coherent, adjacency relations change in a controlled way, and the structure maintains sufficient regularity to support a continuum interpretation. By contrast, if many links concentrate onto increasingly smaller subsets during refinement—so that average incidence grows without bound—then the incidence pattern fails these conditions. This situation corresponds, in the continuum approximation, to the breakdown of geometric interpretability.

**Definition** **6** (Incidence Density Blow-Up)**.** *Let Q be a QHIS and C(Q) its incidence structure (5). For a hodon p∈P, define its incidence degree by*

$$\deg_{I}\left(p\right):=\left|\{\ell \in L:(p,\ell )\in I\}\right|.\;$$

*Let ${\left(P_{n}\right)}_{n\in N}$ be an increasing sequence of operationally defined subsets of P, obtained under refinement. The associated incidence density is*

$$\rho_{I}\left(P_{n}\right):=\frac{1}{|P_{n}|}\sum_{p\in P_{n}}\deg_{I}\left(p\right).\;$$

*We say that $\rho_{I}\left(P_{n}\right)$ blows up if*

$$\lim_{n\to \infty }\rho_{I}\left(P_{n}\right)=+\infty .$$

**Remark** **11** (Locality and the Role of $\rho_{I}$)**.** *The incidence density $\rho_{I}\left(P_{n}\right)$ is fundamentally local: it depends on the incidence pattern within a refining sequence of coarse-grained subsets $\left(P_{n}\right)$ and therefore varies under refinement. In highly symmetric or uniformly biregular configurations—such as the simple examples listed in Table 1—$\rho_{I}\left(P_{n}\right)$ reduces to a multiple of the global ratio |L|/|P|. In general incidence structures, however, the ratio |L|/|P| fails to capture local variations, anisotropies, or refinement behavior. By contrast, $\rho_{I}\left(P_{n}\right)$ remains well defined under refinement and is sensitive to precisely the features that control curvature-like behavior, geometric emergence, and the onset of singular or non-geometric regimes.*

**Remark** **12** (Incidence Blow-Up vs. Discreteness)**.** *Incidence-density blow-up does not violate Axiom 1. By that axiom, every operationally defined subset $P_{n}$ contains only finitely many hodons, and the incidence degree $\deg_{I}\left(p\right)$ is finite for each individual hodon p. Blow-up is therefore not a statement about any single discrete configuration but about the behavior of a refining sequence $\left(P_{n}\right)$ of such subsets under increasing resolution. It signals a failure of the incidence pattern to stabilize in the sense required by the regularity conditions (R1)–(R3), and consequently a breakdown of the manifold-likeness criteria (M1)–(M2). The divergence of $\rho_{I}\left(P_{n}\right)$ thus marks the onset of a non-geometric phase characterized by anomalous combinatorial crowding, rather than any violation of discreteness or finiteness at the fundamental level.*

**Lemma** **1** (Bounded Incidence Density in the Geometric Regime)**.** *Let (P,L,I) be an incidence structure satisfying the regularity and manifold-likeness criteria (R1)–(R3) and (M1)–(M2). Then, for any sequence of subsets $\left(P_{n}\right)$ obtained under refinement, the associated incidence densities $\rho_{I}\left(P_{n}\right)$ are uniformly bounded:*

$$\underset{n}{\sup}\rho_{I}\left(P_{n}\right)<\infty .$$

**Proof.**  Conditions (R1)–(R3) ensure that incidence relations change only locally under refinement and cannot generate unbounded new adjacency. Conditions (M1)–(M2) guarantee that local incidence patterns belong to a fixed finite family of combinatorial models. Together, these imply a uniform bound on the average incidence degree along any refining sequence $\left(P_{n}\right)$. A complete proof reduces to a routine but technically involved combinatorial bounding argument based on this finite family and the associated stability assumptions and introduces no additional conceptual ingredients beyond the stated criteria. □

**Theorem** **4** (Incidence Density Blow-Up and Failure of Geometric Realizability)**.** *Let Q be a QHIS and $Q_{\mathrm{int}}$ the induced interior QHIS on a subset $P_{\mathrm{int}}\subseteq P$. Let $C(Q_{\mathrm{int}})$ denote its incidence structure. If there exists a refining sequence $\left(P_{n}\right)$ with $P_{n}\subseteq P_{\mathrm{int}}$ such that $\lim_{n\to \infty }\rho_{I}\left(P_{n}\right)=+\infty$, then $C(Q_{\mathrm{int}})$ fails at least one of the regularity or manifold-likeness conditions (R1)–(R3), (M1)–(M2). Consequently, by Theorem 2, it is not realizable by the continuum functor F (6) as a smooth Lorentzian region.*

**Proof.**  If the incidence structure satisfied (R1)–(R3) and (M1)–(M2), the preceding lemma would bound $\rho_{I}\left(P_{n}\right)$ uniformly along any refining sequence. The assumption that $\rho_{I}\left(P_{n}\right)\to +\infty$ therefore implies that at least one of the criteria must fail. Theorem 2 then states that failure of these criteria prevents F (6) from assigning any smooth Lorentzian region to the interior structure. □

### 8.2. In Simple Terms

**Figure 2:**
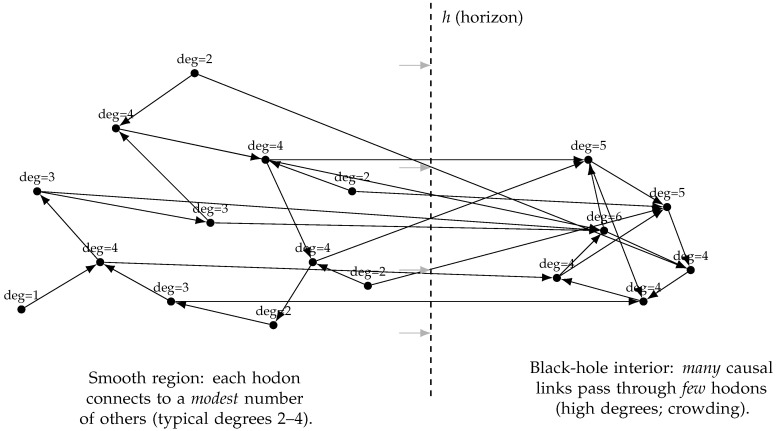
Hodon connectivity and degrees across the horizon. (**Left**): In smooth regions, each hodon has a modest incidence degree (2–4 typical). (**Right**): Inside a black hole, many causal links funnel through fewer hodons, producing high degrees and combinatorial crowding—the discrete analog of focusing and curvature blow-up.

Incidence density measures how many causal links must pass through a collection of hodons as one refines attention to smaller and smaller subsets. In configurations compatible with geometric interpretation, this quantity remains controlled. In interior regions of a black hole, however, the discrete incidence pattern may become increasingly crowded under refinement, causing incidence density to diverge (see Figure 2). When this happens, the incidence structure exits the manifold-like class, and no smooth continuum picture can represent the region. The discrete hodon structure thus signals the breakdown of geometric interpretability long before any continuum invariant diverges.

## 9. Singularity as a Phase Transition in the QHIS

In the QHIS framework, the hodon network Q=(P,L,I,W,H) may undergo a transition from a regime in which its incidence structure supports a continuum interpretation to one in which such an interpretation is no longer possible. This transition reflects a shift from a *geometric phase*—where the combinatorial regularity conditions permit realization by the continuum functor—to a *non-geometric phase* in which the incidence structure fails these conditions. The latter phase corresponds to the onset of singular behavior in the continuum description.

### 9.1. Geometric Phase

From Theorem 2, a QHIS lies in the geometric regime when its underlying incidence structure C(Q)=(P,L,I) satisfies the regularity and manifold-likeness criteria (R1)–(R3) and (M1)–(M2) of Section 3. In this regime, the combinatorial pattern of incidences is sufficiently coherent under refinement for the continuum approximation functor F (6) to assign a smooth Lorentzian spacetime (M,g) whose causal order extends the partial order obtained from classicalization. The QHIS thus behaves, at an emergent level, like a region of classical spacetime.

### 9.2. Phase Transition

As the black hole interior develops, the incidence structure on the interior subset $P_{\mathrm{int}}\subseteq P$ may become increasingly irregular under refinement. In purely combinatorial terms, this is reflected in the concentration of incidences within shrinking subsets or, equivalently, in the divergence of the incidence density $\rho_{I}\left(P_{n}\right)$ along refining sequences. Once this irregularity becomes severe enough to violate one or more of the regularity or manifold-likeness conditions, the interior incidence structure $C(Q_{\mathrm{int}})$ leaves the geometric regime. At this point, it is *non-realizable* by the continuum functor F (6) as any smooth Lorentzian region.

### 9.3. Continuum Interpretation

Under the continuum functor F (6), the transition from realizability to non-realizability corresponds to a failure of smooth extendibility: while the exterior region admits a continuum representation, the interior does not. In continuum terms, this obstruction manifests as geodesic incompleteness. Importantly, this interpretation is *derived*—the discrete framework itself contains no geodesics or curvature, only the incidence relations that determine whether a continuum interpretation can exist.

### 9.4. Comparison

To contextualize this transition, it is informative to compare such non-geometric incidence patterns with familiar finite configurations. Table 1 lists the incidence density $\rho_{I}$ for several representative structures, illustrating how divergence of this quantity correlates with departure from the manifold-like class.

- **Hesse configuration ($\rho_{I}=4$):** Moderate non-uniformity yet still compatible with a geometric realization.
- **Projective plane of order q:** Incidence density increases linearly with *q* but remains uniformly structured, preserving manifold-likeness.
- **Complete graph $K_{n}$:** Exhibits extreme combinatorial crowding. Incidence density grows as n−1, diverging under refinement; such patterns violate the regularity and manifold-likeness criteria and are therefore non-realizable by F (6).
- **Singularity-like configurations:** Even if only a small portion of the hodon set exhibits disproportionately high incidence degree, the divergence in incidence density under refinement signals a transition to the non-geometric phase.

**Remark** **13** (Counterexamples and QHIS Representation)**.** *Classical continuum singularity criteria—such as curvature divergence or geodesic incompleteness—do not always coincide. The QHIS framework naturally accommodates this: global obstructions to realization by F (6) correspond to combinatorial patterns that fail the manifold-likeness criteria, while local crowding may occur without global breakdown. Hence, QHIS captures a broad class of “singularity-like” behaviors within a unified combinatorial language, without relying on continuum constructions.*

### 9.5. Matter and Predictive Scope in Non-Geometric Phases

In classical general relativity, questions about the behavior of matter near a singularity are formulated in geometric terms: one asks how geodesics terminate or how curvature diverges. In the QHIS framework, such geometric notions are not fundamental. Matter is represented instead by quantum information distributed across hodons and along incidence links, and operational observables concern correlations, influence, and informational capacity rather than trajectories or distances.

When the incidence structure lies in the geometric regime, these informational quantities admit an approximate continuum interpretation. However, when the incidence pattern becomes non-manifold-like—failing one or more of the criteria (R1)–(R3) or (M1)–(M2)—the geometric interpretation collapses. In such non-geometric phases, including singularities and early-universe regimes, once sufficient combinatorial crowding develops, the informational degrees of freedom do not disappear; rather, they become encoded in an increasingly dense and highly connected subgraph. In this setting, traditional notions such as “position,” “trajectory,” or “geodesic evolution” lose meaning, but operational notions such as entanglement, influence, and quantum state evolution remain well defined.

**Figure 3:**
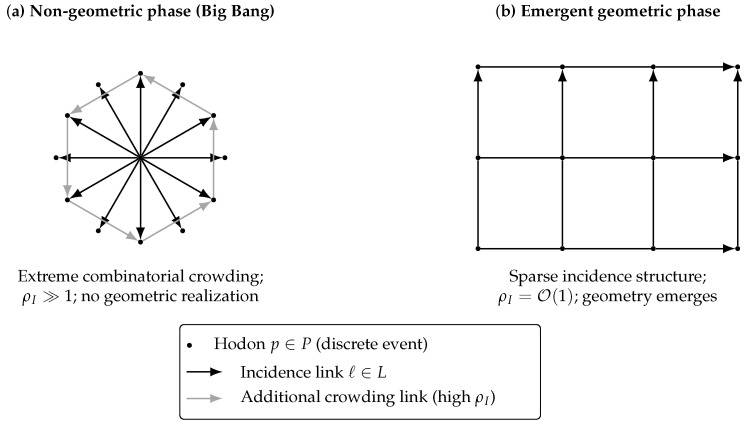
QHIS interpretation of the Big Bang transition. (**a**) An early non-geometric phase with extreme incidence density. (**b**) A reorganized, sparse, manifold-like incidence structure from which spacetime geometry emerges.

From this perspective, questions such as “what is the fate of the infalling observer” or “what emerges from the initial epoch” presuppose geometric notions that are unavailable in the non-geometric phase. The QHIS description instead tracks how informational content is redistributed across hodons and how causal influence becomes increasingly unconstrained. The resulting picture is that of a combinatorial “information cloud” in which almost every elementary event lies in mutual influence with almost every other. In such a regime there is no meaningful separation between events—no operational “space” or “time” in which distinguishable occurrences can be located relative to one another. The emergence of a geometric description after the Big Bang is then interpreted as a phase transition from combinatorial crowding to sparse, coherent connectivity, rather than as matter *and spacetime* emanating from a single point (see Figure 3).

This reframing suggests that the predictive scope of the QHIS framework extends beyond the limits of continuum geometry: even when the latter fails, the quantum informational and causal primitives of the theory remain operationally intact. The transition between non-geometric and geometric phases thus reflects not a breakdown of physics but a change in the descriptive layer appropriate for interpreting the underlying discrete dynamics.

## 10. Reconstruction of the Page Curve in QHIS Language

With the theoretical machinery in place, we now shift from the general structure of the QHIS framework to its concrete applications in black-hole thermodynamics and semiclassical gravity. In each case, the operational and informational organization of the hodon network provides the mechanisms traditionally attributed to quantum fields on curved backgrounds, allowing these phenomena to be reinterpreted in purely causal–informational terms.

Our first application concerns the Page curve. We show how the *content* supplied by Poisson sampling and the *capacity* set by the incidence boundary jointly determine the Page curve (von Neumann entropy) [1,2,20]. This *content vs. capacity* mechanism is the conceptual engine behind the Page-time transition.

### 10.1. Operational Setup (No Geometry Assumed)

Let (P,L,I,W,H) be a QHIS. At coarse-grained time *t*, select two operationally defined disjoint subsets

$$P_{\mathrm{int}}\left(t\right),\;P_{\mathrm{rad}}\left(t\right)\subseteq P\left(t\right),$$

interpreted as “interior” and “radiation.” A finite-resolution selection procedure $S_{\mathrm{rad}}\left(t\right)$ identifies a random subset $P\left(S_{\mathrm{rad}}\left(t\right)\right)\subseteq P_{\mathrm{rad}}\left(t\right)$ of radiation hodons. By Axiom 3,

(9) $$E\left[N_{\mathrm{rad}}\left(t\right)\right]=\alpha \;\mu \left(S_{\mathrm{rad}}\left(t\right)\right),$$

so $N_{\mathrm{rad}}\left(t\right):=N\left(S_{\mathrm{rad}}\left(t\right)\right)$ supplies the subsystem’s *content* (how many quantum units are present).

The *capacity* for transmitting interior–radiation entanglement is determined by the *incidence boundary* $\partial P_{\mathrm{int}}\left(t\right)$ separating $P_{\mathrm{int}}\left(t\right)$ and $P_{\mathrm{rad}}\left(t\right)$—the minimal cut of links whose removal eliminates all positive-propensity paths in both directions—together with the cross-boundary propensities *W* (clause (**viii**) of Axiom 4).

### 10.2. Cross-Boundary Entropy Estimator and Capacity Bound

Define the cross-boundary link set

$$L_{\partial }\left(t\right):=\left\{\ell_{ij}\in L\left(t\right)|v_{i}\in P_{\mathrm{int}}\left(t\right),\;v_{j}\in P_{\mathrm{rad}}\left(t\right)\;\mathrm{or}\;\mathrm{vice}\;\mathrm{versa}\right\}.$$

Each $\ell_{ij}$ carries a propensity $W(\ell_{ij})$ mediating quantum correlation between $H(v_{i})$ and $H(v_{j})$. With a local entanglement contribution $I_{ij}\left(t\right)$, an operational estimator of the radiation entropy is

(10) $$S_{\mathrm{rad}}\left(t\right)\;\approx \;\sum_{\ell \in L_{\partial }\left(t\right)}W\left(\ell \right)\;I_{\ell }\left(t\right),$$

expressed purely in terms of incidence, propensities, and cross-boundary correlations.

By the holographic counting axiom (Axiom 5), interior-accessible information obeys

$$I\left(P_{\mathrm{int}}\left(t\right)\right)\;\leq \;\beta \;\nu \left(\partial P_{\mathrm{int}}\left(t\right)\right),\;$$

so that

$$S_{\mathrm{rad}}\left(t\right)\;\leq \;I\left(P_{\mathrm{int}}\left(t\right)\right)\;\leq \;\beta \;\nu \left(\partial P_{\mathrm{int}}\left(t\right)\right).$$

Thus, *content* grows with $N(S_{\mathrm{rad}}\left(t\right))$, whereas *capacity* is bounded by the incidence-boundary measure and realized through the pattern of *W* on $L_{\partial }\left(t\right)$.

### 10.3. Early-Time Regime (Rising Branch: Content Below Capacity)

According to Equation (9), Poisson sampling increases radiation content. At early times:

- $|L_{\partial }\left(t\right)|$ grows as new quanta are emitted;
- The propensities W(ℓ) vary slowly under coarse graining;
- Typical cross-boundary contributions $I_{ij}\left(t\right)$ remain near their channel maxima.

More radiation hodons provide more opportunities for cross-boundary correlations, and the estimator in Equation (10) therefore rises approximately linearly. Meanwhile, capacity $\beta \;\nu (\partial P_{\mathrm{int}}\left(t\right))$ is far from saturated:

$$\mathrm{Content}\;\mathrm{from}\;\mathrm{Poisson}\;\mathrm{sampling}\;<\;\mathrm{Capacity}\;\mathrm{from}\;\left(W,\nu ,\partial P_{\mathrm{int}}\right)\;⟹\;S_{\mathrm{rad}}\left(t\right)\;\mathrm{rises}.$$

### 10.4. Page Time: Critical Balance of Content and Capacity

As evaporation proceeds, $P_{\mathrm{int}}\left(t\right)$ shrinks while $P_{\mathrm{rad}}\left(t\right)$ grows, and the interior incidence structure becomes increasingly crowded.

**Remark** **14** (Interior Structure Near Page Time)**.** *Before Page time, the interior may remain sufficiently coherent to satisfy the regularity and manifold-likeness criteria (R1)–(R3), (M1)–(M2). Near Page time, global entanglement redistribution increases combinatorial crowding and pushes the interior to the edge of the geometric regime.*

Two trends meet:

**(a)**  **Content growth.** The effective Hilbert-space dimensions
  $$d_{\mathrm{rad}}\left(t\right):=\dim\;\left(\underset{p\in P_{\mathrm{rad}}\left(t\right)}{⨂}H\left(p\right)\right),\;d_{\mathrm{int}}\left(t\right):=\dim\;\left(\underset{p\in P_{\mathrm{int}}\left(t\right)}{⨂}H\left(p\right)\right),$$
  satisfy
  $$\log d_{\mathrm{rad}}\left(t_{\mathrm{Page}}\right)\;\approx \;\log d_{\mathrm{int}}\left(t_{\mathrm{Page}}\right),$$
  so the radiation subsystem now has enough room to encode essentially all correlations shared with the interior.
**(b)**  **Capacity limitation.** The boundary $\partial P_{\mathrm{int}}\left(t\right)$ bounds the supportable interior–radiation correlations, and as evaporation progresses, the propensities *W* on $L_{\partial }\left(t\right)$ approach a *critical incidence configuration* at which no further independent correlations can cross. This fixes the effective maximal capacity through $\nu (\partial P_{\mathrm{int}}\left(t\right))$.
**(c)**  **Page time as equilibrium.** Page time occurs when the cross-boundary estimator, i.e. the weighted sum in Equation (10), first meets the capacity permitted by $\partial P_{\mathrm{int}}\left(t\right)$:
  $$\mathrm{Content}\;\mathrm{from}\;\mathrm{Poisson}\;\mathrm{sampling}\;\approx \;\mathrm{Capacity}\;\mathrm{from}\;\left(W,\nu ,\partial P_{\mathrm{int}}\right)\;⟹\;S_{\mathrm{rad}}\left(t\right)\;\mathrm{peaks}.$$

### 10.5. Late-Time Regime: Capacity Dominates and the Interior Purifies

For $t>t_{\mathrm{Page}}$, interior incidence exits the geometric regime (violation of (R1)–(R3) and/or (M1)–(M2)), and $L_{\partial }\left(t\right)$ can support no new independent correlations. Instead, cross-boundary influence re-routes and purifies existing correlations. Poisson sampling may still enlarge $P_{\mathrm{rad}}\left(t\right)$, and we recall that $N_{\mathrm{rad}}\left(t\right)=\left|P_{\mathrm{rad}}\left(t\right)\right|$, but the incidence boundary’s capacity has already been exhausted:

$$\mathrm{Content}\;\mathrm{from}\;\mathrm{Poisson}\;\mathrm{sampling}\;>\;\mathrm{Capacity}\;\mathrm{from}\;\left(W,\nu ,\partial P_{\mathrm{int}}\right)\;⟹\;S_{\mathrm{rad}}\left(t\right)\;\mathrm{declines}.\;$$

With no further independent correlations able to cross the boundary, the radiation subsystem enters a purification regime: its entropy steadily decreases as cross-boundary propagation serves only to disentangle and redirect previously shared correlations. By global unitarity of the QHIS evolution,

$$S_{\mathrm{rad}}\left(t\right)⟶0,$$

reflecting the complete transfer of correlations to radiation and the operational disappearance of the interior subsystem.

### 10.6. One-Sentence Synthesis

Poisson sampling determines *how many* radiation hodons are present (content), while the cross-boundary propensities *W* together with the incidence-boundary measure ν bound *how much* entanglement can flow (capacity); their competition generates the Page curve, and their balance defines Page time.

## 11. Hawking Thermality from the QHIS Framework

Just as the Page curve analysis revealed how information flow could be reinterpreted in causal and informational terms within the QHIS framework, we now turn to the thermal aspect of black-hole physics. In semiclassical gravity, Hawking radiation is traditionally derived from quantum fields propagating on a fixed curved background. In the QHIS description, however, thermal behavior must arise from variations in the underlying incidence-density profile of the hodon network. The transition from information balance (Page curve) to the thermal behavior of Hawking radiation (hereinafter referred to as Hawking thermality) is natural: both depend on how causal influence is redistributed across a horizon-scale boundary.

To make this precise, we introduce the notion of a *critical layer*—the QHIS counterpart of a black-hole horizon. The critical layer is defined by a sharp gradient in the radial incidence density, signaling a substantial reorganization in the flow of causal influence across the network. In the classical geometric regime, where coarse-grained quantities vary smoothly enough for the continuum functor F to apply, this layer corresponds to the location at which the effective redshift diverges in the continuum approximation.

**Remark** **15** (Local vs. Global Behavior at the Horizon)**.** *The critical layer does not conflict with the equivalence principle: locally, each hodon and its immediate neighborhood follow the same causal and informational rules, and a freely falling observer encounters no discontinuity when crossing the horizon. The horizon’s distinctive role in QHIS therefore reflects a global property of the incidence structure rather than any local physical anomaly or violation of smoothness along infalling worldlines.*

With this structure in hand, we now show how Hawking thermality emerges directly from the incidence-density gradient near the critical layer.

### 11.1. Incidence Density Under the Continuum Approximation

For a coarse-grained subset of hodons P(r) that, under the continuum approximation (functor F (6)), is interpreted as an effective radial shell of radius *r*, the *radial incidence density* is defined as

$$\rho_{I}\left(r\right)\;:=\;\frac{1}{\left|P(r)\right|}\sum_{p\in P(r)}\deg_{I}\left(p\right).$$

Here, the parameter *r* does not denote a fundamental geometric radius; it is an emergent label associated with the coarse-grained organization of hodons and becomes geometric only under the action of F.

In the classical geometric regime (Theorem 2), the continuum approximation functor acts on coarse-grained profiles of the form $r\mapsto \rho_{I}\left(r\right)$ to produce smooth radial functions. For static, spherically symmetric configurations, this gives

(11) $$f\left(r\right)\;=\;F\;\left(\rho_{I}\right)\left(r\right),$$

where f(r) is the lapse function of the emergent metric

$$ds^{2}=-f\left(r\right)\;dt^{2}+f{\left(r\right)}^{-1}dr^{2}+r^{2}d\Omega^{2}.$$

Equation (11) reflects that both $\rho_{I}\left(r\right)$ and f(r) capture the same operational structure of causal influence. In continuum general relativity, f(r) encodes gravitational redshift, the tilt of light cones, and the location of the horizon. In the QHIS framework, $\rho_{I}\left(r\right)$ characterizes the strength of causal influence across coarse-grained shells and measures how rapidly correlations attenuate or concentrate radially. Thus, whenever the incidence structure becomes sufficiently regular to admit a continuum description, the functor F must match the discrete causal profile to its continuum counterpart.

### 11.2. Surface Gravity from Incidence-Density Gradients

Since F maps discrete radial profiles to smooth continuum functions and is smooth on such profiles in the geometric regime, differentiation commutes with F at leading order:

(12) $$\frac{d}{dr}\;F\left(\rho_{I}\right)\left(r\right)\;=\;F\;\left(\rho_{I}^{\prime }\right)\left(r\right),\;$$

where $\rho_{I}^{\prime }\left(r\right)$ denotes the coarse-grained radial derivative of $\rho_{I}\left(r\right)$, and $F(\rho_{I}^{\prime })$ denotes the continuum image of the derivative function $r\mapsto \rho_{I}^{\prime }\left(r\right)$.

Applying (12) to (11) gives

(13) $$f^{\prime }\left(r\right)\;=\;F\;\left(\rho_{I}^{\prime }\right)\left(r\right).$$

Let $r_{h}$ denote the horizon radius, defined by $f(r_{h})=0$. Evaluating (13) at $r=r_{h}$,

(14) $$f^{\prime }\left(r_{h}\right)\;=\;\left(F(\rho_{I}^{\prime })\right)\left(r_{h}\right).$$

In the continuum theory, the surface gravity is

(15) $$\kappa \;=\;\frac{1}{2}f^{\prime }\left(r_{h}\right).$$

Combining (14) and (15) yields the QHIS expression

(16) $$\kappa \;=\;\frac{1}{2}\;\left(F(\rho_{I}^{\prime })\right)\left(r_{h}\right).$$

Thus, κ is determined by the continuum image of the incidence-density gradient at the critical layer. Operationally, $\rho_{I}^{\prime }\left(r_{h}\right)$ quantifies the sharp reorganization of the causal structure near the horizon.

### 11.3. Hawking Thermality from the Discrete-to-Continuum Map

In the continuum description, a stationary horizon that emits a thermal spectrum is assigned a temperature via the standard relation

(17) $$T_{H}\;=\;\frac{\kappa }{2\pi }.$$

This formula does not itself establish thermality; rather, it quantifies the temperature associated with a spectrum already known to be thermal. Substituting (16) into (17) yields the corresponding QHIS expression

(18) $$T_{H}\;=\;\frac{1}{4\pi }\;\left(F(\rho_{I}^{\prime })\right)\left(r_{h}\right).$$

**Figure 4:**
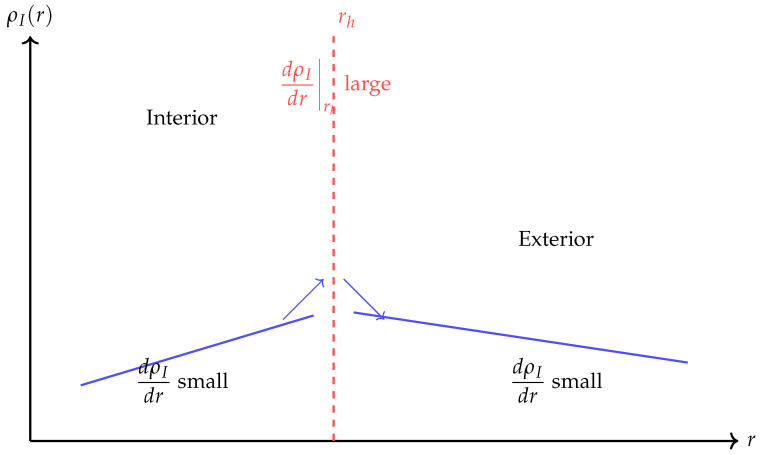
Incidence density profile $\rho_{I}\left(r\right)$ across the horizon. The QHIS framework describes both exterior and interior regions in terms of the incidence density $\rho_{I}\left(r\right)$, independently of any specific metric background. The portion of the profile shown for $r<r_{h}$ is therefore schematic: it represents discrete structural behavior of the hodon network (combinatorial crowding and the growth of $\rho_{I}\left(r\right)$) rather than any metric quantity. The slow rise of $\rho_{I}\left(r\right)$ for $r<r_{h}$ does not depict the singular core; instead, it reflects an effective, coarse-grained incidence density within the manifold-like interior region, excluding the non-geometric core where incidence density becomes extreme and radial localization ceases to be meaningful.

Equation (18) shows that the continuum temperature is the geometric image, under the functor F, of the discrete incidence-density gradient at the critical layer (see Figure 4). No metric structure is assumed at the fundamental level; the thermality of the emitted radiation originates entirely from the behavior of the incidence-density profile, while the continuum relation (18) translates this thermality into a temperature once the continuum description applies.

### 11.4. Generalization to Rotating or Charged Black Holes

The QHIS account of Hawking thermality relies on two structural ingredients: (i) the presence of a critical incidence layer whose incidence-density gradient encodes the near-horizon redshift behavior, and (ii) the identification (13) between this discrete gradient and its continuum image under F. Neither ingredient requires spherical symmetry, so the same reasoning extends naturally to stationary black holes, including Kerr (rotating) and Reissner–Nordström (charged) solutions.

In stationary spacetimes, the event horizon is generated by a Killing vector $\chi^{a}$ that becomes null on the horizon. The associated surface gravity is defined by

(19) $$\kappa \;=\;\frac{1}{2}\;\nabla^{a}\left(\chi^{b}\chi_{b}\right){|}_{r=r_{h}},$$

and takes the standard Kerr or Reissner–Nordström values [21,22]. Under the continuum approximation, the functor F maps the discrete incidence-density gradient at the critical layer, $\rho_{I}^{\prime }\left(r_{h}\right)$, to the geometric quantity $\nabla^{a}\left(\chi^{b}\chi_{b}\right){|}_{r=r_{h}}$ appearing in (19). Thus, the universal relation

$$T_{H}=\frac{\hbar \;\kappa }{2\pi k_{B}c}$$

is recovered directly in the QHIS framework: the continuum temperature reflects the continuum image of the discrete incidence-density gradient, with κ taking whichever stationary value applies. No modification of the QHIS axioms is required; the essential inputs are the discrete incidence-density profile near the horizon and its stationary continuum image under F, which together reproduce the familiar surface-gravity law for the Hawking temperature [3] across Schwarzschild, Kerr, and Reissner–Nordström black holes alike.

### 11.5. Role of the Hilbert-Space Assignment in the Emergence of Hawking Temperature

The preceding analysis shows that the near-horizon thermal behavior of a black hole arises, in the QHIS framework, from variations in the underlying incidence-density profile of the probabilistically causal graph (4). These variations determine how influence propagates across the incidence boundary, how cross-boundary correlations form, and how operational coarse-graining produces an approximately thermal distribution of emitted radiation. None of these structural features requires geometric input; the operational analog of a horizon is encoded entirely by the incidence boundary $\partial P_{\mathrm{int}}\left(t\right)$, and the onset of thermal behavior follows from the combinatorial pattern of influence and correlation across that boundary.

This structural origin of thermality, however, must be distinguished from the definition of a *temperature*. Temperature is a property of a quantum state on a Hilbert space, not merely of a weighted directed graph (P,L,I,W). In the QHIS formalism, the quantum degrees of freedom needed for this interpretation are supplied by the Hilbert-space assignment H (2).

More concretely, assigning a temperature to the radiation requires:

- A density operator $\rho_{\mathrm{rad}}\left(t\right)$ on a tensor-product Hilbert space $H_{\mathrm{rad}}\left(t\right)$,
  $$H_{\mathrm{rad}}\left(t\right)=\left(\underset{p\in P_{\mathrm{rad}}\left(t\right)}{⨂}H\left(p\right)\right)\;$$
- A computation of its von Neumann entropy $S\left(\rho_{\mathrm{rad}}\right)=S_{\mathrm{rad}}\left(t\right)$ (10);
- A comparison of $\rho_{\mathrm{rad}}$ to a Gibbs state of the form $e^{-H/T}$ for some Hamiltonian *H* acting on $H_{\mathrm{rad}}$;
- A meaningful notion of quantum modes or energy levels.

None of these ingredients is available from (P,L,I,W) alone. Although the incidence structure determines the kinematic *pattern* of thermality—by controlling influence propagation and boundary capacity—the quantitative assignment of a Hawking temperature rests entirely on the Hilbert spaces and their associated operators.

In summary, the probabilistically causal graph (4) provides the geometry-free structural origin of Hawking thermality, but the Hilbert-space assignment (2) is indispensable for defining a Hawking temperature. The complete QHIS tuple (P,L,I,W,H) is therefore essential for recovering the thermodynamic content of Hawking radiation.

## 12. QHIS and Semiclassical Paradoxes

Having seen how the QHIS framework reproduces both the Page curve and the Hawking thermality from purely causal–informational principles, we now turn to the paradoxes that arise when semiclassical expectations are pushed to their limits.

Traditional discussions of black-hole information often highlight apparent contradictions between unitarity, smooth horizons, and semiclassical quantum field theory. In the QHIS description, however, these tensions take on a different character: they reflect incompatible assumptions about how information and causal influence are distributed across the underlying hodon network. By analyzing the incidence structure near a critical layer, we can reinterpret familiar paradoxes as statements about the organization, flow, and redistribution of informational degrees of freedom rather than about competing geometric pictures.

### 12.1. Black-Hole Dissolution in a Violent Burst

In semiclassical general relativity, the Hawking temperature grows as $T_{H}\propto 1/M$, so that the evaporation accelerates and the temperature diverges as the black-hole mass *M* approaches the Planck scale. Thus, according to the semiclassical prediction, the black hole ends in a violent burst. Critically, this behavior presumes that the continuum description remains valid throughout the evaporation process, allowing the use of the surface-gravity relation (17) down to arbitrarily small horizon radii.

In the QHIS framework, however, the continuum temperature $T_{H}$ is not fundamental but the geometric image, under the approximation functor F, of a discrete incidence-density gradient $\rho_{I}^{\prime }\left(r_{h}\right)$ at the critical layer. The thermal behavior of the emitted radiation originates entirely from the combinatorial structure of the hodon network, while the continuum expression (18) merely assigns a temperature to that thermality once the incidence structure lies in the geometric regime.

After the Page time, the relevant incidence patterns on the interior side of the critical layer transition into a non-geometric phase, marked by combinatorial crowding and the failure of the geometric-regime criteria (R1)–(R3) and (M1)–(M2). In this regime, the continuum functor F can no longer realize the incidence structure as a smooth Lorentzian region, and the notion of a continuum horizon radius $r_{h}$ ceases to apply. Consequently, Equation (18) is no longer valid, and the assignment of a Hawking temperature breaks down.

Thus, the divergent-temperature endpoint predicted by semiclassical gravity reflects not a physical blow-up but the extrapolation of the continuum formula beyond its domain of validity. In the QHIS description, the evaporation process ceases to admit a continuum interpretation before any such divergence occurs. After Page time, Hawking thermality remains (as a structural property), but Hawking temperature—being a continuum scalar—no longer exists.

### 12.2. The Firewall Paradox

The AMPS firewall paradox [8] arises from the apparent incompatibility of three statements: (i) Hawking radiation is globally pure; (ii) effective field theory (EFT) holds outside a microscopic neighborhood of the horizon; and (iii) an infalling observer experiences no discontinuity when crossing the horizon. In geometry-first frameworks, these assumptions refer to properties of quantum fields on a fixed spacetime manifold. Combined, they create tension: global purity requires late-time radiation to be entangled with earlier radiation, while EFT and smoothness for the infaller require short-range entanglement across the horizon; these conditions cannot all hold simultaneously.

**Figure 5:**
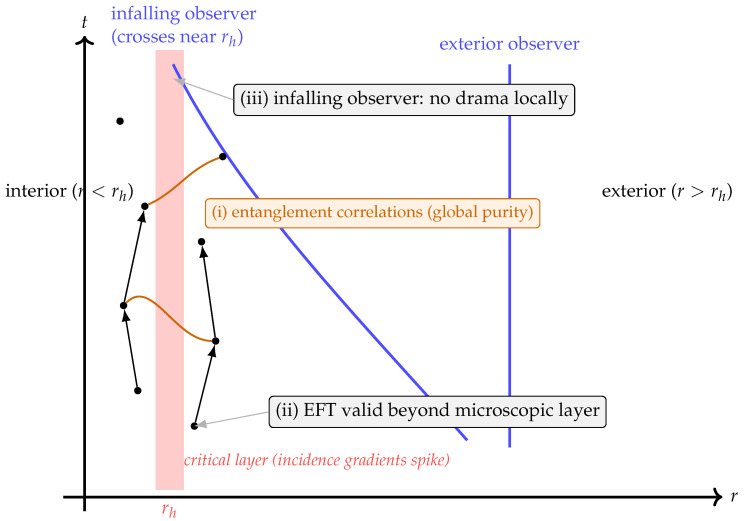
Three conditions and the critical layer in QHIS. Hodons (black dots) represent discrete events p∈P, while incidence lines represent causal relations ℓ∈L between them.

In the QHIS framework, these assumptions are reformulated operationally in terms of hodons, incidence, weights, and informational capacity (see Figure 5). Since geometry is not fundamental, no contradiction arises at the level of the discrete substrate.

- **Global Purity:** Each hodon and link carries a finite-dimensional Hilbert space, and the global QHIS state ρ is represented by a density operator on the tensor-product Hilbert space $H_{\mathrm{global}}$ (3). The evolution of ρ is unitary in the sense of Definition 1, meaning that it respects the directed structure of *L* and introduces no influence beyond the permitted link incidences. Consequently, the global state of the entire hodon network remains pure at all times. Observers who access only operationally defined subsets of *P* may therefore perceive mixed states, but this mixedness reflects coarse-graining rather than any loss of purity in the full system.
- **Critical Layer and EFT:** Instead of a geometric horizon, the QHIS features a critical layer at which the incidence pattern undergoes rapid reorganization, typically signaled by a sharp rise in incidence density (Section 8). Outside this narrow region, the incidence structure may satisfy the regularity and manifold-likeness conditions (R1)–(R3) and (M1)–(M2), allowing EFT to emerge as a coarse-grained description via the continuum functor F (6). Crucially, no smooth geometry is assumed at the fundamental level; EFT appears only where the incidence structure admits a stable continuum approximation.
- **Smooth Experience for Infallers:** Locally, each hodon and its adjacent links obey the same operational rules regardless of whether the global incidence pattern is in the geometric regime. No special discontinuity appears in the local Hilbert assignments (2) at the incidence boundary. Smooth experience for infallers is therefore an emergent property of locally regular informational behavior, not a geometric condition imposed fundamentally.

#### 12.2.1. Entanglement Handling

In the QHIS, entanglement is a structural feature of correlations among Hilbert spaces attached to hodons and links. Near the incidence boundary, such correlations can be redistributed without violating unitarity. The holographic counting bound (Axiom 5) limits the amount of accessible information in terms of the abstract boundary measure ν(∂R) rather than a geometric area. This flexibility permits global purity to be maintained while allowing effective field-theoretic behavior to emerge outside the critical layer, without requiring any “firewall-like” disruption of near-boundary correlations.

#### 12.2.2. Relation to ER = EPR

The ER = EPR conjecture [23] interprets entanglement as a geometric connection via wormholes, embedding quantum correlations within a geometry-first ontology. In contrast, the hodon framework takes an information-first perspective: hodons, probabilistic causal links, and Hilbert assignments are fundamental, and geometry emerges only when the incidence structure meets the regularity and manifold-likeness conditions permitting realization by F (6). Both viewpoints agree that entanglement underlies connectivity, but QHIS implements this principle without invoking wormholes or a prior manifold. Instead, it encodes structure through discrete incidence, causal propensity, and informational capacity constraints, offering an operational resolution of the firewall paradox.

### 12.3. The Duplication Experiment

The traditional duplication experiment presupposes that a single observer can both reconstruct information from Hawking radiation and later compare it with an interior copy after crossing the horizon. In the QHIS framework, this presupposition fails at the operational level. An observer is represented by a finite operational subset of hodons, and the information accessible to that observer is bounded by the holographic counting bound (Axiom 5). Collecting sufficient Hawking radiation to reconstruct interior information already saturates the informational capacity permitted by the observer’s incidence boundary. When the same observer subsequently enters the black hole, the relevant incidence structure may transition into a non-geometric phase, characterized by combinatorial crowding and loss of manifold-likeness. In that phase, the operational distinction between “stored radiation information,” “interior degrees of freedom,” and “comparison operations” ceases to be well defined. As a result, there exists no single operationally coherent QHIS subsystem in which both purported copies of the information are simultaneously accessible and comparable. Information duplication is therefore not an observable phenomenon in QHIS, and black-hole complementarity is preserved as an emergent consequence of operational finiteness, holographic capacity, and the phase-dependent validity of geometric description.

### 12.4. The “Enormous Interior Volume” Problem

Semiclassical general relativity predicts that the interior of an evaporating black hole may possess an enormous and rapidly growing spatial volume, even as the horizon shrinks. This creates an apparent paradox: such a large interior seems capable of storing an arbitrarily large amount of information, in tension with the holographic bound and with unitary evaporation. In the QHIS framework, this tension dissolves because interior “volume” exists only when the incidence structure lies in the geometric regime. Once the interior becomes sufficiently crowded to violate the regularity and manifold-likeness criteria, the continuum functor F (6) no longer assigns a meaningful spatial geometry to that subset. An analogous resolution applies to bag-of-gold geometries [24,25,26], whose enormous semiclassical interiors far exceed the information that can cross their narrow throats. In QHIS, such bag-of-gold interiors correspond to incidence structures that have left the geometric regime; their apparent volume growth reflects a transition to a non-geometric phase rather than the emergence of new information-bearing capacity. Informational content remains governed strictly by the holographic bound on the incidence boundary, not by any semiclassical notion of interior size.

### 12.5. Immunity of QHIS to Wigner-Friend No-Go Arguments

Recent extensions of the Wigner’s friend scenario to black-hole evaporation have produced no-go theorems suggesting that no post-quantum theory can simultaneously maintain unitarity, smooth horizons, and observer-independent outcomes [27]. These arguments rely on assumptions that implicitly presume a globally valid continuum spacetime and a universal Hilbert-space description accessible to all observers. The QHIS framework evades these no-go results because it does not satisfy these assumptions in the first place.

#### 12.5.1. No Universal Super-Observer

Wigner-friend constructions presuppose the existence of a “super-observer” who can model the full physical system unitarily, including the internal states of other observers. QHIS forbids such access: every observer is tied to an *operationally defined subset* of hodons, and the causal-consistency condition (Definition 1) enforces strictly local unitary evolution compatible with the directed-link structure. No observer can act globally on $H_{\mathrm{global}}$, preventing the nonlocal operations required by the no-go theorems.

#### 12.5.2. No Absolute Event Identity

Wigner-friend paradoxes rely on the Absoluteness of Observed Events (AOE)—that all observers must assign the same definite outcome to every measurement. QHIS assigns outcomes only relative to *selection procedures* S, and different observers may have access to distinct operational subsets. Event identity is therefore operational rather than absolute, undermining the logical structure of the no-go arguments, which require the simultaneous comparison of observers’ outcome assignments.

#### 12.5.3. No Shared Geometric Background

The no-go arguments assume that all observers can be embedded in a *single* smooth spacetime. In QHIS, geometry is phase-dependent: only when the incidence structure satisfies (R1)–(R3) and (M1)–(M2) does a continuum spacetime exist. After Page time, interior regions typically become *non-geometric*. Thus, the assumption of a unified geometric arena for multiple observers simply does not hold.

#### 12.5.4. No Simultaneous Access to Interior Partner Modes

Wigner-friend no-go constructions require an observer to compare Hawking radiation with interior partner modes. QHIS prevents this: cross-boundary entanglement flow is bounded by the incidence-boundary capacity $\nu (\partial P_{\mathrm{int}})$, and the interior loses its geometric and subsystem meaning in the non-geometric phase. There is no operational sense in which observers can jointly access interior and exterior modes.

Taken together, these features show that the assumptions underlying the Wigner-friend no-go theorems do not apply to QHIS. The paradoxes are avoided not through modification of quantum theory but because their operational premises have no counterpart in the QHIS description.

### 12.6. Synopsis

Collectively, these observations show that the semiclassical paradoxes of black-hole physics—the “violent end point”, the firewall argument, the duplication experiment, the apparent growth of interior volume, and recent Wigner-friend-style no-go arguments—arise only when geometric or observer-independent reasoning is applied beyond its operational domain. In the QHIS framework, geometry is not fundamental but emerges only when the incidence structure satisfies the regularity and manifold-likeness criteria. Once this geometric regime fails, questions that presuppose a smooth spacetime—such as whether evaporation accelerates exponentially near the Planck scale, whether interior and exterior copies of information can coexist, how large the interior volume becomes, or whether different observers must assign consistent absolute outcomes—lose operational meaning.

In particular, the strengthened Wigner-friend no-go arguments rely on assumptions, such as the existence of a universal super-observer, the absoluteness of measurement outcomes, and a single shared geometric background, that have no counterpart in the QHIS formalism. Because observers in QHIS are restricted to operationally defined subsets, the incidence structure may enter a non-geometric phase, and the interior subsystem ceases to have a continuum interpretation, the premises required for such no-go theorems cannot be formulated. Thus, the apparent contradictions dissolve not through modifications of quantum mechanics or appeals to complementarity, but because their operational assumptions lie outside the phase-dependent domain where a continuum or global observer description applies.

QHIS therefore reinterprets the apparent tensions of semiclassical gravity as artifacts of extending continuum or observer-independent reasoning too far. The framework provides a consistent, information-theoretic account valid across both geometric and non-geometric phases, in which black-hole paradoxes cease to arise once the operational scope of each phase is respected.

## 13. A Physical Narrative of the Four Phases of the Hodon Network

The formal results developed in this work reveal that a Quantum Hodonic Influence Structure does not describe a single kind of “spacetime” but rather admits several distinct phases depending on the balance between quantum degrees of freedom and the combinatorial regularity of the underlying incidence relations. These phases provide a useful physical narrative for understanding how classical geometry emerges, how it fails, and how quantum structure persists beyond the limits of smooth spacetime.

### 13.1. Classical Geometric Phase

In the classical geometric phase, the QHIS sits simultaneously in the classicalization regime and in the geometric regime. Probabilistic and quantum features are suppressed, yielding a deterministic incidence structure, and the regularity and manifold-likeness conditions are satisfied. As shown by Theorems 1 and 2, such a structure is faithfully realizable as a smooth Lorentzian spacetime. In this phase the hodons behave like classical events, and causal links admit interpretation as familiar causal curves. It is in this regime that ordinary relativistic physics takes place.

### 13.2. Classical Non-Geometric Phase

In the classical non-geometric phase, the quantum degrees of freedom are still collapsed, but the incidence structure fails one or more of the geometric-regime criteria. The system is therefore deterministic but not manifold-like. This corresponds exactly to the situation analyzed in Theorem 3: the incidence structure is well defined, yet no smooth Lorentzian region can approximate it. This is the discrete counterpart of a classical singularity, where geometry breaks down not because of any divergence in local fields but because the incidence pattern no longer admits a geometric realization.

### 13.3. Quantum Geometric Phase

In the quantum geometric phase, the hodon network carries genuine quantum correlations and probabilistic influence relations, while its incidence structure continues to satisfy the geometric-regime criteria. The continuum approximation functor therefore assigns a smooth spacetime to this phase, albeit one modulated by quantum fluctuations and entanglement. This is the regime in which semiclassical gravity, early-universe quantum geometry, or black-hole evaporation prior to the Page time naturally reside: geometry remains valid as an emergent description, but it coexists with a nontrivial quantum structure.

As an illustration, the emergence of the Hawking temperature is localized to the quantum geometric phase rather than the classical geometric phase because:

- The incidence structure satisfies the geometric-regime criteria (R1)–(R3) and (M1)–(M2);
- The continuum approximation functor F assigns a valid Lorentzian spacetime interpretation;
- But the Hilbert-space degrees of freedom remain active and entangled across the incidence boundary.

### 13.4. Quantum Non-Geometric Phase

The quantum non-geometric phase is the fully non-classical and non-manifold-like regime. Here, the network exhibits significant quantum correlations while the incidence pattern violates the geometric-regime conditions. As a result, no smooth Lorentzian geometry can approximate the structure. This is the quantum analog of a singularity: the network remains perfectly well defined at the level of quantum causal and informational degrees of freedom, but the geometric description fails entirely. Situations such as the late-stage interior of an evaporating black hole or the earliest pre-geometric moments of cosmology naturally fall into this category.

### 13.5. Summary

**Figure 6:**
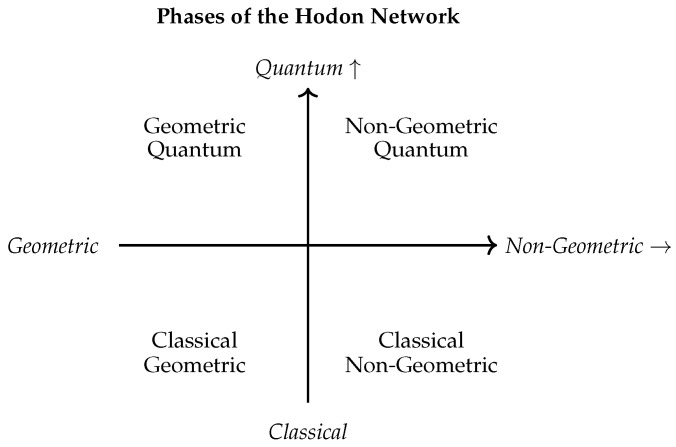
Four phases of the hodon network: classical vs. quantum and geometric vs. non-geometric.

The four phases provide a coherent physical picture of how spacetime can behave within the QHIS. Geometry appears only in the phases where the incidence structure satisfies the appropriate regularity conditions, and it disappears when those conditions fail. Classically, this corresponds to the emergence or breakdown of smooth spacetime; quantum mechanically, it highlights how causal and informational structure remains well defined even where geometry no longer applies. Figure 6 summarizes this landscape.

## 14. QHIS Cosmology

### 14.1. Status of Non-Geometric Components Within QHIS

The QHIS formalism permits regions of the global incidence structure that fail to satisfy the regularity and manifold-likeness criteria required for a continuum spacetime interpretation. This permissiveness is not a mere mathematical curiosity: within QHIS, non-geometric phases acquire clear physical meaning whenever geometric realizability breaks down. Such phases play an essential role in the framework’s account of the Big Bang as a geometric phase transition, the internal structure of black holes, the Page curve, and the dissolution of semiclassical paradoxes. In this sense, non-geometric regimes are not optional extensions of the theory but physically significant phases of the same underlying causal–informational structure.

At the same time, QHIS remains deliberately agnostic about the global prevalence of non-geometric sectors. The framework specifies the structural conditions under which geometry emerges or fails, without asserting that such failures must occur universally or that additional non-geometric components must exist beyond those required to account for known phenomena. Non-geometric phases are therefore physically meaningful wherever they arise, while their broader cosmological distribution remains an open question rather than a fixed prediction of the framework.

### 14.2. Phase Structure and Emergent Geometry

The QHIS framework naturally leads to a cosmological picture in which geometry is not a universal background but a contingent, phase-dependent feature of a deeper informational substrate. Rather than presupposing a globally defined spacetime manifold, QHIS begins with a discrete incidence structure of hodons and directed causal influence relations. Smooth Lorentzian geometry emerges only in those regions where the incidence pattern satisfies the regularity and manifold-likeness criteria required for the continuum approximation functor F to apply.

Within this interpretation, the Big Bang is not understood as a curvature singularity embedded in spacetime but as a geometric nucleation event: a transition from a densely connected, non-manifold-like regime to a phase in which continuum spacetime becomes meaningful. The pre-geometric regime is characterized by high incidence density, irregular connectivity, and the absence of any valid continuum description. The onset of manifold-likeness marks the emergence of classical spacetime, with Lorentzian geometry appearing as an effective descriptor of the underlying discrete dynamics. Classical cosmology thus describes the interior of a geometric phase rather than the totality of the Universe.

Once geometry has emerged, its applicability remains local and contingent. QHIS permits regions in which the geometric-regime conditions fail—such as black-hole interiors or extreme early-universe domains—to revert to non-geometric behavior. In these regimes, the continuum functor ceases to apply, and classical notions such as metric curvature, geodesic completeness, or spacetime volume lose operational meaning. Singularities are therefore reinterpreted as transitions out of the geometric phase, rather than as pathological divergences within geometry itself.

More broadly, the global incidence structure may admit additional non-geometric components that never undergo geometric nucleation or that remain causally inaccessible from within a given geometric sector. While QHIS does not posit the existence of such components as a necessary feature of cosmology, it provides a coherent language in which such possibilities are physically admissible. Geometry is thus treated as a phase property of the incidence structure rather than as a universal attribute of reality.

Cosmology within QHIS may be viewed as the study of phase dynamics of a discrete causal–informational substrate. The evolution of the Universe is governed not by the expansion of a pre-existing manifold but by the emergence, persistence, and breakdown of geometric phases. The Big Bang corresponds to the onset of geometric order; black holes represent localized returns to non-geometric behavior; and the long-term fate of the Universe may involve a transition to regimes in which geometry no longer provides an adequate description. This phase-structured cosmology offers a unified conceptual framework for emergent spacetime, singularity resolution, and the limits of semiclassical reasoning, while remaining open to a broader non-geometric global structure.

### 14.3. Relation to Major Approaches to Quantum Gravity and Standard Cosmological Models

The cosmological picture suggested by the QHIS framework shares important motivations with several major approaches to quantum gravity, while differing from each in its structural commitments and interpretive stance. It is therefore useful to situate QHIS cosmology within this broader landscape.

**Causal set theory** [15,28,29,30,31] provides the closest structural analog, as both approaches begin from a discrete, order-theoretic substrate. In causal set theory, spacetime emerges when a locally finite partial order approximates a Lorentzian manifold. QHIS generalizes this picture by enriching the incidence structure with probabilistic causal weights and finite-dimensional Hilbert spaces, thereby integrating quantum informational degrees of freedom directly into the fundamental substrate. Moreover, QHIS does not require the underlying structure to be globally a partial order: acyclicity and manifold-likeness are phase-dependent properties rather than universal constraints. This allows QHIS to accommodate non-geometric regimes, such as the pre-geometric Big Bang or black-hole interiors, without interpreting them as failures of the fundamental theory.

**Loop quantum cosmology** [32,33,34] replaces the classical Big Bang singularity with a quantum bounce within a quantized geometric phase space. While this approach modifies the dynamics of geometry, it retains a fundamentally geometric ontology. QHIS differs conceptually in that the pre-geometric regime is not a quantum geometry but a non-geometric informational phase in which no continuum description is meaningful. The Big Bang is interpreted not as a bounce but as the nucleation of geometric order from a combinatorial substrate, and QHIS does not require geometry to re-emerge after a breakdown.

**Holographic and entanglement-based approaches** [35,36,37,38] emphasize the role of quantum information in reconstructing spacetime geometry. QHIS aligns with this informational perspective but departs in its treatment of geometry as a non-universal phase. Rather than assuming the existence of a bulk spacetime whenever a dual description applies, QHIS allows the continuum approximation to fail in a controlled and physically meaningful manner. Boundaries and holographic capacity constraints arise from incidence relations rather than from pre-existing geometric surfaces.

Taken together, these comparisons highlight the distinctive position of QHIS cosmology. Like causal set theory, it begins from a discrete causal substrate; like loop quantum cosmology, it replaces classical singularities with non-classical phases; and like holographic approaches, it grounds geometry in informational structure. Its defining departure lies in treating geometry as a phase-dependent, non-universal construct and in allowing non-geometric regimes to be physically meaningful rather than merely transitional.

## 15. Observational Prospects for QHIS Cosmology

Although the QHIS framework is fundamentally discrete and operational rather than field-theoretic or geometric, it leads to a set of observational prospects that are increasingly accessible through modern astrophysical, cosmological, and laboratory platforms. These prospects should be understood as *structural signatures* of phase-dependent geometry rather than as sharp quantitative predictions tied to a specific dynamical model. In QHIS, observations probe where and how the continuum approximation breaks down and how information flow is constrained in regimes where geometry ceases to be a valid descriptor.

### 15.1. Black-Hole Phenomenology and Horizon-Scale Observations

Black holes provide the most direct observational window into the phase structure of spacetime suggested by QHIS. The framework identifies black-hole interiors and near-horizon regions as domains in which the manifold-likeness conditions may fail while the exterior remains well described by classical geometry. This motivates several observational avenues.

#### 15.1.1. Gravitational-Wave Spectroscopy

Post-merger ringdown signals probe the near-horizon structure of compact objects. QHIS suggests that geometricity may fail prior to classical singularity formation, potentially leading to structural deviations from the Kerr ringdown spectrum, including late-time modifications, horizon-scale modulations, or echo-like features associated with non-geometric interior structure. Such effects would signal a breakdown of the continuum approximation in regimes traditionally modeled by smooth geometry.

#### 15.1.2. Black-Hole Shadow and Horizon Imaging

High-resolution horizon-scale imaging, such as that provided by the Event Horizon Telescope and future interferometric arrays, allows tests of whether the effective horizon behaves as a smooth geometric surface. QHIS permits small-scale irregularities in the effective horizon structure, deviations from ideal photon-ring morphology, or features correlated with incidence-density gradients rather than local curvature. Although such signatures are expected to be subtle, next-generation observations may place constraints on non-geometric deviations from classical horizon structure.

#### 15.1.3. Hawking-like Emission and Analog Systems

Because QHIS derives thermality from incidence-density gradients rather than from near-horizon redshift in a smooth background, deviations from perfect thermality become natural observables. In analog gravity systems—where entanglement flow and mode conversion are experimentally accessible—such deviations may provide indirect evidence for the underlying informational mechanism proposed by QHIS.

### 15.2. Early-Universe Signatures and Primordial Correlations

Interpreting the Big Bang as a geometric nucleation event implies that the earliest Universe was non-geometric. This pre-geometric phase may leave imprints inaccessible to manifold-based cosmological models but potentially visible in large-scale observables.

#### 15.2.1. Cosmic Microwave Background Anomalies

A non-geometric pre-Big-Bang phase may produce statistical features inconsistent with standard inflationary assumptions, including large-angle anomalies, departures from statistical isotropy, or non-Gaussian correlation structures not captured by smooth spacetime evolution. Such features would reflect the transition from a densely connected, irregular incidence structure to a geometric phase.

#### 15.2.2. Primordial Power Spectrum Deviations

If the onset of geometricity was gradual rather than instantaneous, the primordial power spectrum may exhibit scale-dependent departures from simple power-law behavior, suppressed correlations at the largest scales, or features associated with the advancing front of geometric order. These effects would encode information about the phase boundary rather than about geometric expansion alone.

#### 15.2.3. Primordial Gravitational Waves

A pre-geometric regime may also alter the expected spectrum of primordial gravitational waves. QHIS suggests the possibility of suppressed tensor modes, modified polarization patterns, or non-standard correlations reflecting incidence-based rather than geometric dynamics. While not unique to QHIS, such signatures would motivate models in which early cosmology is not fully geometric.

### 15.3. Laboratory Analogs and Controlled Quantum Systems

Because QHIS grounds gravitational phenomena in informational and combinatorial constraints, laboratory systems that simulate entanglement flow, horizons, or dissipative boundaries offer valuable testing grounds.

#### 15.3.1. Analog Hawking Radiation

Experiments in Bose–Einstein condensates, nonlinear optical systems, and fluid analogs can probe deviations from thermality tied to incidence-density gradients and capacity constraints. QHIS predicts that entanglement saturation and emission properties are governed by boundary capacity rather than by geometric area, a distinction that can be tested directly in controlled analog environments.

#### 15.3.2. Quantum Simulators and Evaporating Subsystems

Quantum computing platforms and many-body simulators can model evaporating subsystems with tunable boundary connectivity. QHIS suggests that Page-curve behavior, entanglement saturation, and transitions to non-geometric phases depend on informational flow limits rather than on geometric notions of size. Such simulations provide a direct test of the informational principles underlying the framework.

### 15.4. Holographic Capacity Tests Beyond Geometry

QHIS interprets holographic bounds as arising from incidence boundaries rather than from geometric surfaces. This perspective suggests observational prospects wherever geometric area and informational capacity diverge, including evaporating black holes, wormhole-like configurations, or cosmological horizons with non-standard causal structure. If informational capacity tracks incidence structure more closely than geometric area, such systems would favor an incidence-based interpretation of gravitational entropy.

### 15.5. Outlook

The observational prospects of QHIS cosmology span astrophysical observations, cosmological surveys, analog gravity experiments, and quantum simulations. Rather than requiring modifications of Einstein’s equations or the introduction of new particles, QHIS motivates tests of where and how geometric descriptions fail and how information flows in such regimes. As experimental and observational capabilities advance, these structural signatures provide a pathway for distinguishing phase-dependent emergent geometry from models in which spacetime is assumed to be universally valid. In this sense, QHIS cosmology is not merely a conceptual alternative to manifold-based models but a framework with concrete and increasingly accessible avenues for empirical exploration.

## 16. Conclusions

The thermodynamic behavior of evaporating black holes brings together several interrelated entropy phenomena, including Hawking thermality, the growth of entanglement between a black hole and its radiation, and the requirement of entropy purification implied by unitarity. Reconciling these features has long motivated investigations into how information is distributed and recovered in black-hole evaporation, giving rise to the Page curve as a central diagnostic of entropy evolution.

In this work we have developed the Quantum Hodon Incidence Structure (QHIS) as a discrete, operational framework in which spacetime geometry is neither fundamental nor universal but emerges as a phase-dependent description of a deeper causal–informational substrate. By grounding gravitational phenomena in incidence relations, probabilistic influence, and local quantum degrees of freedom, QHIS provides a unified language for discussing spacetime emergence, thermality, information flow, and the breakdown of classical geometry in extreme regimes.

Within this framework, several longstanding problems in black-hole physics and cosmology acquire a common explanation. Hawking thermality arises from horizon-scale gradients of incidence density, with temperature appearing only as a derived continuum image. The Page curve emerges from capacity constraints on entanglement flow across incidence boundaries, independent of semiclassical assumptions about interior geometry. Black-hole interiors themselves are interpreted as localized returns to non-geometric behavior, dissolving the apparent inconsistencies associated with smooth horizons, duplication, and firewall arguments. More broadly, the Big Bang is reinterpreted not as a curvature singularity but as a geometric nucleation event marking the onset of manifold-likeness within a previously non-geometric regime.

From a cosmological perspective, QHIS suggests that the observable Universe corresponds to a geometric phase embedded within a larger incidence structure that may include non-geometric components or causally inaccessible regions. While the framework does not assert the existence of such components as a necessary feature of reality, it renders them conceptually coherent and physically admissible. Cosmology in QHIS may therefore be viewed as the study of phase dynamics within a discrete relational substrate, rather than the evolution of a single, globally valid spacetime manifold. Standard cosmological models are recovered as effective descriptions within the geometric phase, without being elevated to universal status.

An important aspect of the QHIS approach is its compatibility with observational inquiry. Although fundamentally pre-geometric, the framework motivates a range of structural signatures associated with the boundaries and breakdown of geometricity, including horizon-scale deviations in black-hole phenomenology, early-Universe correlation features, analog-gravity experiments, and quantum-simulation tests of informational capacity bounds. These prospects illustrate that phase-dependent emergent geometry is not merely a conceptual possibility but a framework with growing empirical relevance.

More broadly, QHIS illustrates how causal structure and quantum information can be treated as coequal primitives from which geometry arises, rather than as quantities defined *within* spacetime. This shift allows classical reasoning to be recovered where it is applicable while making explicit the limits of geometric description. In doing so, QHIS offers a consistent and flexible foundation for exploring cosmology and gravity in regimes where manifold-based assumptions are no longer adequate.

The framework presented here is not intended as a complete theory of quantum gravity but as a structural platform within which emergent spacetime, thermality, and information flow can be analyzed in a unified and operational manner. By clarifying when geometry applies, why it fails, and how physical processes persist beyond its domain, QHIS opens new avenues for understanding the global structure of the Universe and the role of information in gravitational physics. As observational and experimental capabilities continue to advance, the phase-structured cosmology suggested by QHIS provides a fertile ground for further theoretical development and empirical exploration.

## Figures and Tables

**Table 1:** Incidence density $\rho_{I}$ for selected configurations.

Configuration	|P|	|L|	Points/Line	Lines/Point	$\rho_{I}$
Fano plane	7	7	3	3	3
Desargues configuration	10	10	3	3	3
Hesse configuration	9	12	3	4	4
Projective plane of order *q*	$q^{2}+q+1$	$q^{2}+q+1$	q+1	q+1	q+1
Complete graph $K_{n}$	*n*	n2	2	n−1	n−1

## Data Availability

The original contributions presented in the study are included in the article, further inquiries can be directed to the corresponding author.

## References

[1] Page D.N. (1993). Information in Black Hole Radiation. Phys. Rev. Lett..

[2] Page D.N., Mann R.B., McLenaghan R.G. (1994). Black Hole Information. Proceedings of the 5th Canadian Conference on General Relativity and Relativistic Astrophysics, Waterloo, ON, Canada, 13–15 May 1994.

[3] Hawking S.W. (1975). Particle Creation by Black Holes. Commun. Math. Phys..

[4] Birrell N.D., Davies P.C.W. (1984). Quantum Fields in Curved Space.

[5] Bousso R. (2002). The Holographic Principle. Rev. Mod. Phys..

[6] Swingle B. (2012). Entanglement Renormalization and Holography. Phys. Rev. D.

[7] Susskind L., Thorlacius L., Uglum J. (1993). The Stretched Horizon and Black Hole Complementarity. Phys. Rev. D.

[8] Ahmed A., Donald M., Joseph P., Sully J. (2013). Black Holes: Complementarity or Firewalls?. arXiv.

[9] Bekenstein J.D. (1973). Black Holes and Entropy. Phys. Rev. D.

[10] Ryu S., Takayanagi T. (2006). Holographic Derivation of Entanglement Entropy from AdS/CFT. Phys. Rev. Lett..

[11] Hubeny V.E., Rangamani M., Takayanagi T. (2007). A Covariant Holographic Entanglement Entropy Proposal. J. High Energ. Phys..

[12] Almheiri A., Engelhardt N., Marolf D., Maxfield H. (2019). The entropy of bulk quantum fields and the entanglement wedge of an evaporating black hole. J. High Energ. Phys..

[13] Akersa C., Penington G. (2021). Leading order corrections to the quantum extremal surface prescription. J. High Energ. Phys..

[14] Bombelli L., Lee J., Meyer D., Sorkin R.D. (1987). Space-Time as a Causal Set. Phys. Rev. Lett..

[15] Sorkin R.D. (2005). Causal Sets: Discrete Gravity. Lectures on Quantum Gravity.

[16] Oreshkov O., Costa F., Brukner C. (2012). Quantum correlations with no causal order. Nat. Commun..

[17] Costa F., Shrapnel S. (2016). Quantum causal modelling. New J. Phys..

[18] Bolotin A. (2025). Finite geometry and black hole stability: Embedding discrete space into classical manifolds. Acad. Quantum.

[19] Bolotin A. (2025). Discreteness as ontology: A hodon-based approach to dark matter. Europhys. Lett..

[20] Harlow D. (2016). Jerusalem Lectures on Black Holes and Quantum Information. Rev. Mod. Phys..

[21] Chruściel P.T., Costa J.L., Heusler M. (2012). Stationary Black Holes: Uniqueness and Beyond. Living Rev. Relativ..

[22] Raychaudhuri A.K. (1955). Relativistic Cosmology. I. Phys. Rev..

[23] Juan M., Susskind L. (2013). Cool horizons for entangled black holes. arXiv.

[24] Chen P., Ong Y.C., Yeom D.H. (2015). Black hole remnants and the information loss paradox. Phys. Rep..

[25] Almheiri A., Hartman T., Maldacena J., Shaghoulian E., Tajdini A. (2021). The entropy of Hawking radiation. Rev. Mod. Phys..

[26] Raju S. (2022). Lessons from the information paradox. Phys. Rep..

[27] Walleghem L. (2026). Wigner’s friend’s black hole adventure: An argument for complementarity?. arXiv.

[28] Fotini M., Lee S. (1997). Causal evolution of spin networks. Nucl. Phys. B.

[29] Markopoulou F. (2000). Quantum causal histories. Class. Quant. Grav..

[30] Fotini M., Lee S. (1998). Quantum geometry with intrinsic local causality. Phys. Rev. D.

[31] Oreshkov O., Giarmatzi C. (2016). Causal and causally separable processes. New J. Phys..

[32] Rovelli C. (2004). Quantum Gravity.

[33] Perez A. (2013). The Spin-Foam Approach to Quantum Gravity. Living Rev. Relativ..

[34] Gambini R., Olmedo J., Pullin J. (2014). Quantum black holes in loop quantum gravity. Class. Quantum Gravity.

[35] Harlow D. (2017). The Ryu-Takayanagi Formula from Quantum Error Correction. Commun. Math. Phys..

[36] Dong X., Harlow D., Wall A.C. (2016). Reconstruction of Bulk Operators within the Entanglement Wedge in Gauge-Gravity Duality. Phys. Rev. Lett..

[37] Pastawski F., Yoshida B., Harlow D., Preskill J. (2015). Holographic Quantum Error-Correcting Codes: Toy Models for the Bulk/Boundary Correspondence. J. High Energy Phys..

[38] Giddings S.B. (2013). Nonviolent nonlocality. Phys. Rev. D.

